# The m^6^A methylation perturbs the Hoogsteen pairing-guided incorporation of an oxidized nucleotide†
†Electronic supplementary information (ESI) available. See DOI: 10.1039/c7sc02340e



**DOI:** 10.1039/c7sc02340e

**Published:** 2017-07-06

**Authors:** Shaoru Wang, Yanyan Song, Yafen Wang, Xin Li, Boshi Fu, Yinong Liu, Jiaqi Wang, Lai Wei, Tian Tian, Xiang Zhou

**Affiliations:** a College of Chemistry and Molecular Sciences , Key Laboratory of Biomedical Polymers of Ministry of Education , Wuhan University , Wuhan , Hubei 430072 , P. R. China . Email: xzhou@whu.edu.cn ; Email: ttian@whu.edu.cn ; Fax: +86-27-68756663 ; Tel: +86-27-68756663

## Abstract

Natural nucleic acid bases can form Watson–Crick (WC) or Hoogsteen (HG) base pairs. Importantly, 8-oxo-2′-deoxyguanosine (8-oxo-dG) in DNA or 8-oxo-dG 5′-triphosphate (8-oxo-dGTP) favors a *syn* conformation because of the steric repulsion between O8 and O4′ of the deoxyribose ring. 8-oxo-dGTP can be incorporated into DNA opposite the templating adenine (A) using HG pairing as the dominant mechanism. Both RNA and DNA can be methylated at the N6 position of A to form *N* ^6^-methyladenine (m^6^A). It has been found that certain viral infections may trigger an increase in the production of both 8-oxo-dGTP and m^6^A. The current study aims to systematically explore the effects of m^6^A methylation on HG base pairs and the consequent nucleotide incorporation. Our thermodynamic melting study shows that the m^6^A·8-oxo-dG is significantly less stable than the A·8-oxo-dG base pair in the paired region of a DNA duplex. Moreover, we have used pre-steady-state kinetics to examine the incorporation of 8-oxo-dGTP opposite m^6^A relative to A by a variety of reverse transcriptase (RT) enzymes and DNA polymerase (DNA pol) enzymes such as the human immunodeficiency virus type 1 (HIV-1) RT and human DNA pol β. The results demonstrate that all of these enzymes incorporate 8-oxo-dGTP less efficiently opposite m^6^A relative to A. Considering the steric bulk of the purine–purine pair between 8-oxo-dG and A, m^6^A methylation may affect the HG pairing to a great extent. Hence, it will be unfavorable to incorporate 8-oxo-dGTP into the growing strand opposite m^6^A. Moreover, the impeded incorporation of 8-oxo-dGTP opposite m^6^A has been extended to determine m^6^A at pre-defined positions in human rRNA. Our study may provide new insights into the roles of m^6^A in reducing the mutagenic potential of cellular 8-oxo-dGTP.

## Abstract 2

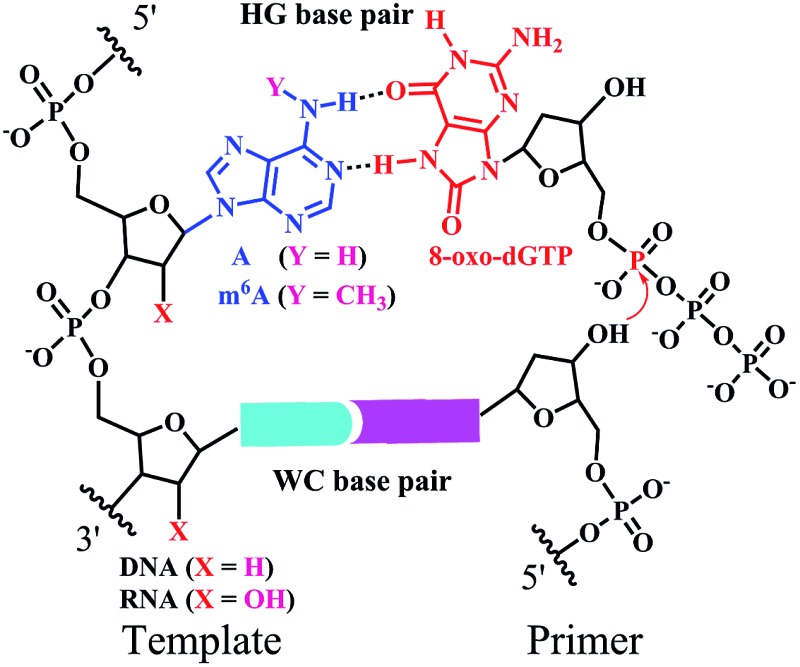
This study describes the structural implications and properties of m^6^A in reducing the incorporation of an oxidized nucleotide into DNA.

## Introduction

Two nucleobases, one on each strand, are held together by hydrogen bonds and form base pairs.^1^ The Watson–Crick (WC) pairings of guanine (G) with cytosine (C) or adenine (A) with thymine (T) are crucial in the formation of double-stranded DNA and in the storage of genetic information (Fig. S1a†).^2^ Nucleic acid bases can also form Hoogsteen (HG) base pairs in the major groove of a WC base-paired DNA duplex (Fig. S1b†).^3^ Many of the properties of HG pairings are quite different from those of WC pairings. Typically, the HG geometry presents a smaller C1′–C1′ distance and a larger angle between the two glycosidic bonds than the WC one.^4^ In particular, one base has been rotated by 180° relative to the other one in reversed HG base pairs. Although HG base pairs are not considered to be common structures, they may be critical for expanding the structural complexity of DNA.

**Fig. 1:**
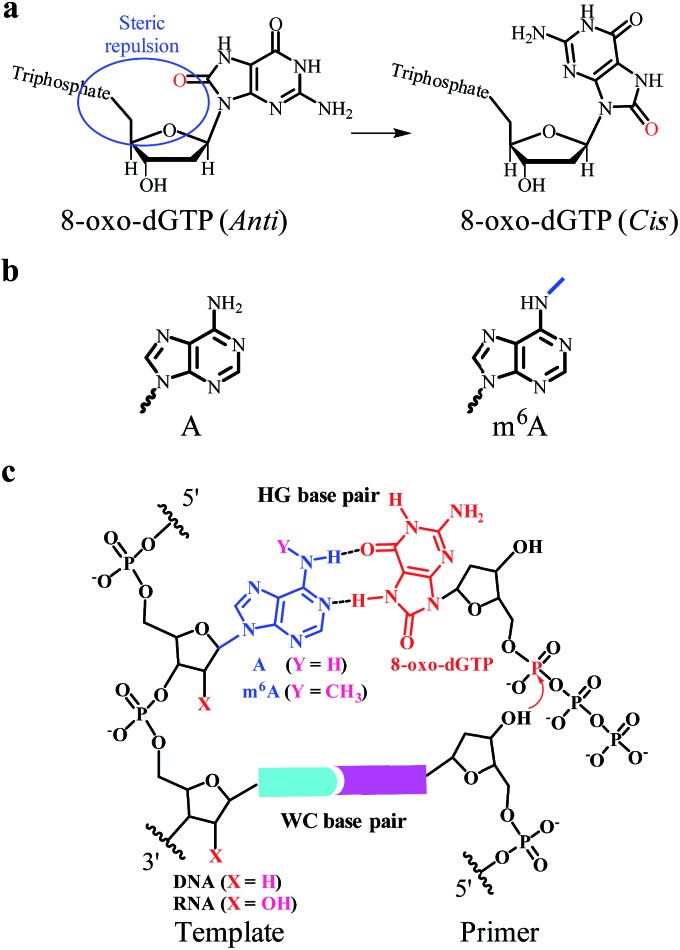
The incorporation of 8-oxo-dGTP opposite m^6^A or A. (a) The different conformations (*anti* or *cis*) of 8-oxo-dGTP. (b) The chemical structure of A or m^6^A. (c) The m^6^A methylation perturbs the HG pairing-guided incorporation of 8-oxo-dGTP.

Earlier studies demonstrate that certain chemical lesions can enhance the stability of HG base pairs.^5^ During cellular metabolic processes, oxidative stress leads to the production of 8-oxo-2′-deoxyguanosine (8-oxo-dG) in DNA and 8-oxo-dG 5′-triphosphate (8-oxo-dGTP in Fig. 1a) in the cellular nucleotide pools.^6^ Increased levels of 8-oxo-dGTP are found to have large effects on boosting mutagenesis in cells.^7^ It has been recognized that 8-oxo-dGTP can form the WC pairing with C, but also the HG pairing with A.^8^ Moreover, the efficiency of 8-oxo-dGTP incorporation opposite the templating A is even higher than that opposite C, resulting in the occurrence of a transversion mutation.^8b,9^ This probably arises from the steric repulsion between the O8 and Pα of 8-oxo-dGTP in the anti-conformation (left part in Fig. 1a).^10^ To circumvent this situation, the base of 8-oxo-dGTP needs to rotate by roughly 180° to the *syn*-conformation (right part in Fig. 1a), thus facilitating the HG pairing with A.^8*b*^ In this manner, 8-oxo-dGTP can be incorporated into DNA opposite A using HG pairing as the dominant mechanism.^11^

Natural nucleic acids contain a large number of naturally-occurring modifications in order to achieve structural and functional specificities.^12^ Importantly, both RNA and DNA contain *N* ^6^-methyladenine (m^6^A in Fig. 1b), which is formed by methylation of A at its exocyclic amino group. Although m^6^A has been identified for a few decades, this modification received little attention in eukaryotes until the discovery of two human m^6^A demethylases, the fat mass and obesity-associated protein (FTO) and α-ketoglutarate-dependent dioxygenase AlkB homolog 5 (ALKBH5).^13^ Earlier studies show that certain viral infections may trigger an increase in the production of 8-oxo-dGTP and m^6^A modification.^14^ It has been found that m^6^A methylation is able to affect the stability and conformation of canonical WC base-paired duplexes and thus impacts the enzymatic properties.^15^ However, there have been no reports demonstrating the effects of m^6^A methylation on HG base pairs and the consequent nucleotide incorporation.

The current study is the first to disclose that the m^6^A:8-oxo-dG is less stable than the A:8-oxo-dG base pair within the paired region of a DNA duplex. We further tested a variety of reverse transcriptase (RT) and DNA polymerase (DNA pol) enzymes, such as human immunodeficiency virus type 1 (HIV-1) RT and human DNA pol β, for their ability to incorporate 8-oxo-dGTP opposite the A/m^6^A residue at a defined position (Fig. 1c). The pre-steady-state single-turnover nucleotide incorporation assay demonstrates that 8-oxo-dGTP is significantly less efficiently incorporated opposite the templating m^6^A than A within the same sequence context. Moreover, we extended our findings to the site-specific detection of m^6^A in either a RNA or DNA context. Our study may provide new insights into the roles of m^6^A methylation in reducing the mutagenic potential of cellular 8-oxo-dGTP.

## Results

### The m^6^A methylation destabilizes the A·8-oxo-dG base pair in the paired region of DNA duplex

Considering the steric bulk of the purine–purine pair between 8-oxo-dG and A, m^6^A methylation may affect this HG pairing interaction to a great extent.^15*a*^ A UV melting study was performed to evaluate the effects of m^6^A modification on the stability of the HG pairing in the paired region of a DNA duplex. We designed and prepared a variety of DNA duplexes with the same sequence except the examined residue (sequences in Tables S1 and S2†). The duplexes consisted of the 13-base pair sequences:

5′-CTGACTXATGCTG-3′

3′-GACTGAYTACGAC-5′

for the corresponding DNA duplex where X = A/m^6^A/C and Y = 8-oxo-dG/T/G.

**Fig. 2:**
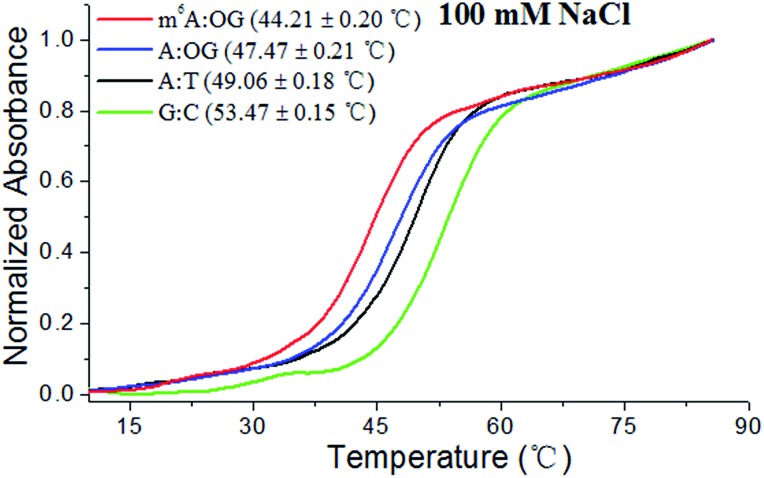
The m^6^A methylation destabilizes HG base pairs in the paired region of DNA duplexes. Representative melting profiles of different duplexes (10 μM) were recorded in 10 mM Tris–HCl buffer (pH 7.0, 100 mM NaCl).

In this investigation, melting curves were recorded at 260 nm and the dissociation of DNA duplexes was monitored. Fig. 2 and S2† show normalized UV melting curves and corresponding melting temperatures of the different duplexes under varied concentrations of NaCl. As expected, m^6^A modification causes a pronounced shift in the melting curve and the *T*_m_ of the ‘m^6^A:OG’ duplex significantly decreased compared to that of the ‘A:OG’ duplex. Moreover, the ‘A:T’ and ‘C:G’ duplexes showed higher *T*_m_ values compared with that of the ‘A:OG’ duplex, probably because of the stronger base pairing. These results showed an evident correlation between the base pairing strength and the stability of the DNA duplex. It is probable that m^6^A modification destabilizes duplex DNA through alleviating the HG pairing. We are therefore encouraged to address the effects of m^6^A modification on the incorporation of 8-oxo-dGTP by RT and DNA pol enzymes.

### RNA m^6^A methylation perturbs the HG pairing-guided incorporation of 8-oxo-dGTP

Earlier studies demonstrated that viral infection may diminish the capacity of the host’s antioxidant system to control oxidative stress.^16^ Importantly, human T cells co-infected with HIV-1 and mycoplasmas have been shown to release hydrogen peroxide,^17^ which can promote the production of 8-oxo-dGTP. This may constitute an important route for base substitution mutations in the host. Moreover, HIV-1 infection can trigger an increase in m^6^A modification of both the viral and host mRNAs.^18^ Upon entry into the host cell, the virally encoded HIV-1 RT reverse transcribes the virus RNA genome into double-stranded DNA. Hence, we were intrigued to study the activities of HIV-1 RT for the incorporation of 8-oxo-dGTP opposite m^6^A relative to A.

**Fig. 3:**
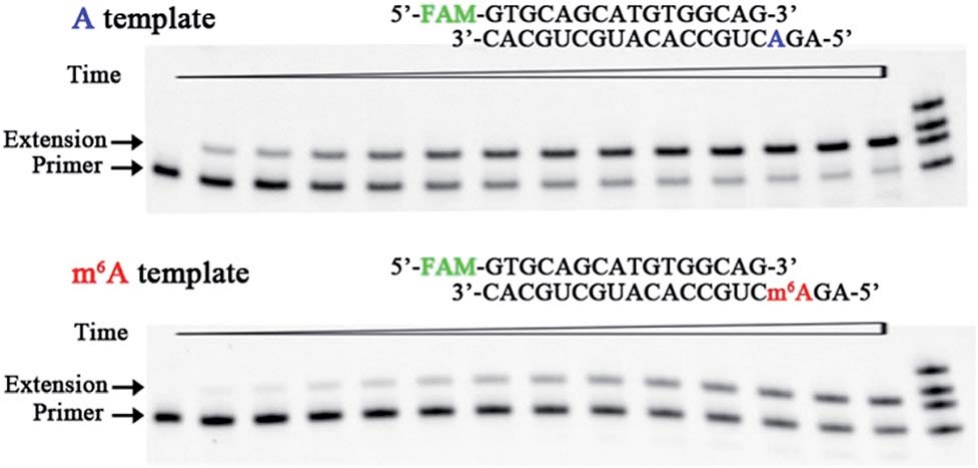
Impeded 8-oxo-dGTP incorporation by HIV-1 RT opposite m^6^A relative to A. Images of representative gels are shown here. Time points are 0, 0.5 min, 1.0 min, 2.0 min, 3.0 min, 4.5 min, 6 min, 8 min, 10 min, 13 min, 16 min, 20 min, 25 min, and 30 min (left to right). Reactions were carried out as described in the ESI† using 50 nM primer/template duplex and 98 nM 8-oxo-dGTP. The oligonucleotides (primer1, primer1 + 1, primer1 + 2 and primer1 + 3 in Table S1†) were used as size markers.

We therefore designed a pair of A/m^6^A templates (RNA1-A and RNA1-m^6^A in Table S1†) with the same sequence, in which the target A/m^6^A site is near to the 5′ end. The extension DNA primer (primer1 in Table S1†) is designed such that its 3′ end lies immediately adjacent to the target residue (A/m^6^A). The incorporation scaffold was set up by assembling the RT enzyme with the primer/template duplex. The first assay is the single-turnover incorporation assay, in which the HIV-1 RT enzyme was allowed to elongate for different incubation times varying from 0.5 min to 30 min. Fig. 3 demonstrates representative data with HIV-1 RT in the presence of 98 nM 8-oxo-dGTP. Importantly, the capacity of 8-oxo-dGTP incorporation was evidently reduced for the ‘m^6^A template’ relative to that of the ‘A template’. Specifically, HIV-1 RT efficiently elongated along RNA1-A and produced an evident ‘extension’ band corresponding to 8-oxo-dGTP incorporation after an incubation of 2 min (lane 4, top gel in Fig. 3), while only a negligible extension was observed for RNA1-m^6^A under the same conditions (lane 4, bottom gel in Fig. 3). HIV-1 RT can bypass the m^6^A site with prolonged incubation (longer than 6 min) indicating that this RNA modification is not an absolute blockage for the HG pairing-guided incorporation of 8-oxo-dGTP (lane 7, bottom gel in Fig. 3).

In the above studies, we used the scaffold 1 with the primer (primer1) ending in –G. Since the 3′ end of the primer lies in the direction in which extension occurs, it is vitally important to examine the primer ending in either –C, –T, or –A. Hence, three new scaffolds (details in Table S1 and Fig. S3†) were assembled for 8-oxo-dGTP incorporation. Time course reactions (0.5 to 30 min) were performed using an excessive enzyme relative to the primer/template duplex. As indicated in Fig. S3,† the extension along the ‘m^6^A template’ was much less efficient than that of the ‘A template’. These results consistently demonstrated the significant effects of RNA m^6^A methylation on impeding the incorporation of 8-oxo-dGTP.

**Fig. 4:**
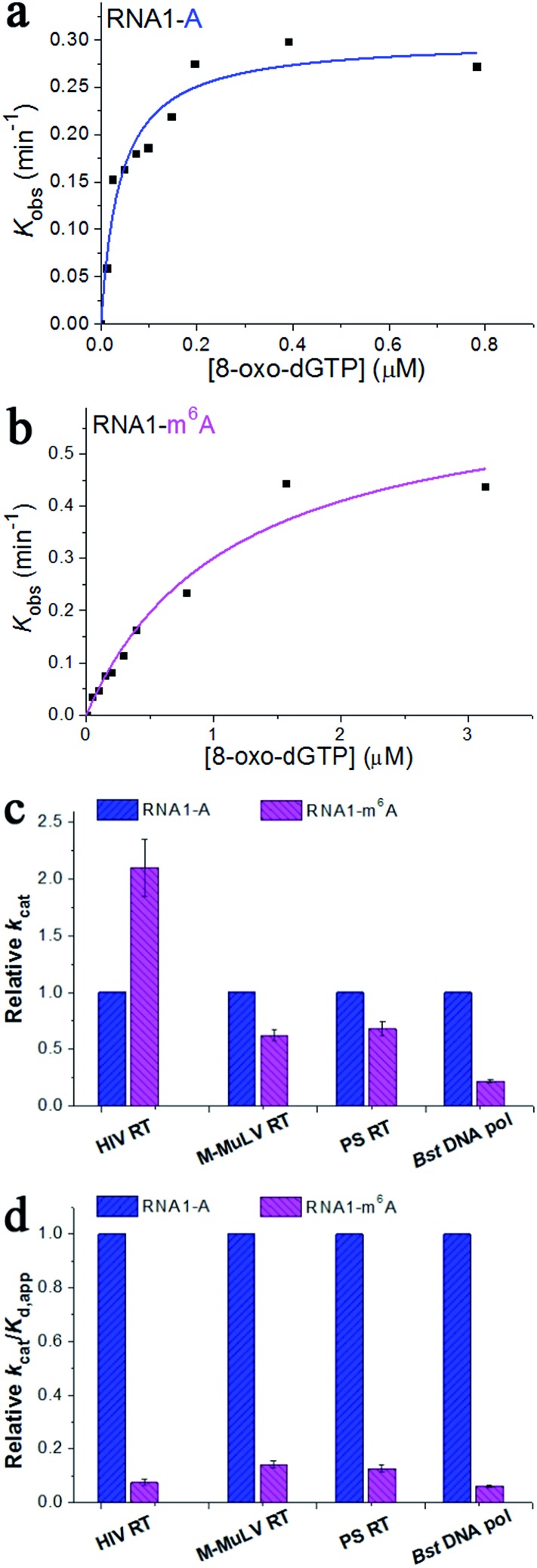
The pre-steady-state kinetics of 8-oxo-dGTP incorporation by HIV-1 RT. (a and b) Representative kinetic fitting curves of 8-oxo-dGTP incorporation opposite m^6^A or A in the RNA template. The 17-nt product was plotted as a function of time and fit to eqn (1) to obtain the reaction rate *k*_obs_. The dependence of *k*_obs_ on 8-oxo-dGTP concentration was fit to eqn (2) in order to obtain *k*_cat_ and *K*_d,app_. (c and d) The relative kinetic constants (*k*_cat_ or *k*_cat_/*K*_d,app_) of HIV-1 RT reverse transcription opposite m^6^A relative to A. All of the kinetic constants are summarized in Table 1.

To gain further insight into the impact of m^6^A methylation, we performed the pre-steady-state single-turnover incorporation assay.^19^ Then, HIV-1 RT was allowed to elongate in the presence of various concentrations of 8-oxo-dGTP and the amount of extended primers was plotted against different incubation times for each examined 8-oxo-dGTP concentration.^20^ This study allows the accurate determination of the kinetic parameters *k*_cat_ (the catalytic rate constant of nucleotide incorporation) and *K*_d,app_ (the apparent nucleotide dissociation constant). The ratio *k*_cat_/*K*_d,app_ defines the measure of catalytic efficiency and substrate specificity. The concentrations of 8-oxo-dGTP were varied from 0.012 to 4.69 μM and a representative range of data with HIV-1 RT are shown in Fig. S4 and S5.† On the basis of the fit (Fig. 4a and b), m^6^A methylation in the RNA template contributes to a 2.1-fold increase in *k*_cat_ (Fig. 4c), a 28.5-fold increase in *K*_d,app_, and a 13.4-fold reduction in the catalytic efficiency (*k*_cat_/*K*_d,app_) with HIV-1 RT (Fig. 4d). This quantitative investigation further confirmed the impact of RNA m^6^A methylation on impeding the incorporation of 8-oxo-dGTP with HIV-1 RT.

The Moloney Murine Leukemia Virus RT (M-MuLV RT) and the ProtoScript® II RT (PS RT) are commonly used to synthesize DNA from RNA in molecular biology.^15b,21^ Recently, the *Bacillus stearothermophilus* DNA polymerase (*Bst* DNA pol) has been found to possess important innate RT activities.^22^ These RT enzymes were also included in the current research. The pre-steady-state single-turnover incorporation assay was performed (Fig. S6–S11†) and the kinetic parameters of 8-oxo-dGTP incorporation opposite A/m^6^A are illustrated in Table 1. Very similar patterns were observed for the M-MuLV RT, PS RT, and *Bst* DNA pol, where the catalytic efficiency (*k*_cat_/*K*_d,app_) was reduced by 7.0-fold, 7.9-fold and 16.2-fold, respectively (Fig. 4d). These results together evidenced an important effect of RNA m^6^A methylation on the incorporation of 8-oxo-dGTP. Additionally, the direct comparison of *k*_cat_/*K*_d,app_ of each enzyme indicates that HIV-1 RT displays the highest activity in the HG pairing-guided incorporation of 8-oxo-dGTP, while *Bst* DNA pol exhibits the highest discrimination between m^6^A and A.

**Table 1:** The impact of m^6^A on the HG pairing-guided incorporation of 8-oxo-dGTP

Enzyme	Template	*k*_cat_, min^–1^	*K*_d,app_, μM	*k*_cat_/*K*_d,app_, μM^–1^ min^–1^	Discrimination^*a*^
HIV RT	RNA1-A	0.30 ± 0.02	0.04 ± 0.01	7.5 ± 0.7	13.4 ± 1.4
	RNA1-m^6^A	0.64 ± 0.07	1.14 ± 0.28	(5.6 ± 0.8) × 10^–1^	
M-MuLV RT	RNA1-A	3.97 ± 0.52	155 ± 30	(2.6 ± 0.4) × 10^–2^	7.0 ± 1.2
	RNA1-m^6^A	2.48 ± 0.17	677 ± 80	(3.7 ± 0.3) × 10^–3^	
ProtoScript® II RT	RNA1-A	0.79 ± 0.045	128 ± 17	(6.2 ± 0.4) × 10^–3^	7.9 ± 0.6
	RNA1-m^6^A	0.54 ± 0.045	695 ± 125	(7.8 ± 0.8) × 10^–4^	
*Bst* DNA pol	RNA1-A	1.10 ± 0.14	151 ± 36	(7.3 ± 1.2) × 10^–3^	16.2 ± 2.8
	RNA1-m^6^A	0.24 ± 0.012	531 ± 46	(4.5 ± 0.2) × 10^–4^	
*Bst* DNA pol	DNA1-A	2.16 ± 0.20	35 ± 6.3	(6.2 ± 0.7) × 10^–2^	18.8 ± 2.2
	DNA1-m^6^A	1.42 ± 0.048	424 ± 27	(3.3 ± 0.1) × 10^–3^	

^*a*^Discrimination = (*k*_cat_/*K*_d,app_)RNA1-A/(*k*_cat_/*K*_d,app_)RNA1-m^6^A or (*k*_cat_/*K*_d,app_)DNA1-A/(*k*_cat_/*K*_d,app_)DNA1-m^6^A.

### DNA m^6^A methylation perturbs the HG pairing-guided incorporation of 8-oxo-dGTP

**Fig. 5:**
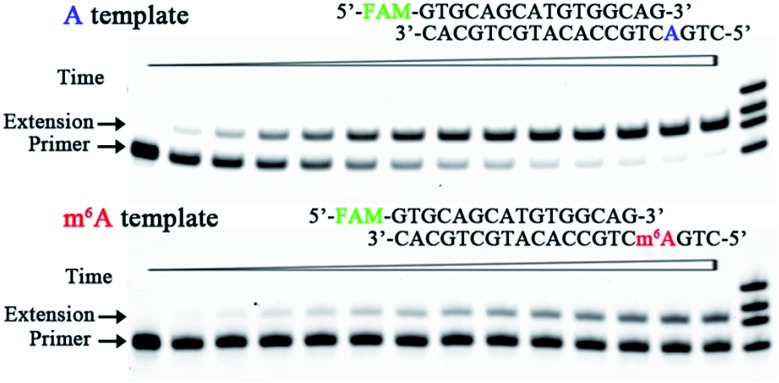
Impeded 8-oxo-dGTP incorporation by human DNA pol β opposite m^6^A relative to A. Images of representative gels are shown here. Time points are 0, 0.5 min, 1.0 min, 2.0 min, 3.0 min, 4.5 min, 6 min, 8 min, 10 min, 13 min, 16 min, 20 min, 25 min, and 30 min (left to right). Reactions were carried out as described in the ESI† using 50 nM primer/template duplex and 25 μM 8-oxo-dGTP. The oligonucleotides (primer1, primer1 + 1, primer1 + 2, and primer1 + 3) were used as size markers.

In human cells reactive oxygen species (ROS) induce many forms of DNA damage, and human DNA pol β performs the base excision repair required for DNA maintenance and replication.^23^ Human DNA pol β is an error-prone enzyme,^24^ which belongs to the eukaryotic-type family X of DNA pol. Since m^6^A has been found in the genomic DNA of various eukaryotes,^25^ we were tempted to use human DNA pol β and test whether DNA m^6^A can perturb the incorporation of 8-oxo-dGTP. A pair of the methylated and unmethylated DNA templates (DNA1-A and DNA1-m^6^A in Table S1†) and primer1 were used to test the ability of human DNA pol β to use 8-oxo-dGTP for incorporation opposite m^6^A. Fig. 5 and S12† illustrate representative data with human DNA pol β in the presence of various concentrations of 8-oxo-dGTP. On the basis of the results in Fig. 5, human DNA pol β extended the 16-nt DNA primer along DNA1-A to predominantly the 17-nt product after an incubation for 4.5 min. In contrast, only minimal 8-oxo-dGTP incorporation was seen opposite m^6^A under the same conditions. Hence, human DNA pol β does incorporate 8-oxo-dGTP less efficiently opposite m^6^A relative to A.

To test the universality of our findings, we further investigated some other scaffolds in which the primer ends in either –C, –T, or –A. The corresponding results showed that DNA m^6^A methylation consistently reduced the HG pairing-guided incorporation of 8-oxo-dGTP by human DNA pol β (Fig. S13†).

**Fig. 6:**
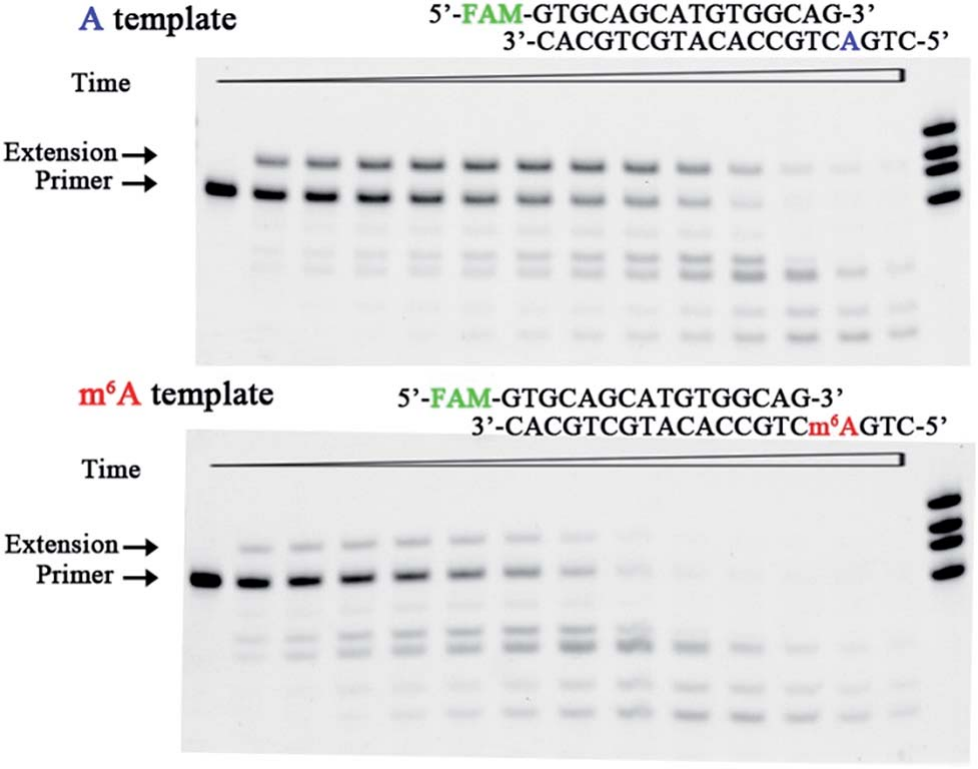
Impeded 8-oxo-dGTP incorporation by φ29 DNA pol opposite m^6^A relative to A. Images of representative gels are shown here. Time points are 0, 0.5 min, 1.0 min, 2.0 min, 3.0 min, 4.5 min, 6 min, 8 min, 10 min, 13 min, 16 min, 20 min, 25 min, and 30 min (left to right). The bands of the shorter ladder than the primer indicate the products from exonucleolytic degradation. Reactions were carried out as described in the ESI† using 50 nM primer/template duplex and 50 μM 8-oxo-dGTP. The oligonucleotides (primer1, primer1 + 1, primer1 + 2, and primer1 + 3) were used as size markers.

The bacteriophage φ29 DNA pol is a protein-primed DNA replicase belonging to the eukaryotic-type family B of DNA pol.^26^ It is known to possess both 5′–3′ polymerization and 3′–5′ exonuclease activities. This study assessed the effect of DNA m^6^A on the ability of φ29 DNA pol to incorporate 8-oxo-dGTP. Fig. 6 illustrates the representative data with φ29 DNA pol in the presence of 50 μM 8-oxo-dGTP. On the basis of these results, DNA m^6^A methylation does reduce the φ29 DNA pol-catalysed incorporation of 8-oxo-dGTP in the complementary strand. The exonuclease activity was demonstrated by the ability of φ29 DNA pol to degrade the primer and form shorter DNA fragments (Fig. 6). Much more rapid primer degradation was observed for the methylated scaffold (DNA1-m^6^A) under the assay conditions. The experiments described above clearly demonstrate that 8-oxo-dGTP is less efficiently incorporated by φ29 DNA pol opposite m^6^A relative to A (Fig. 6).

**Fig. 7:**
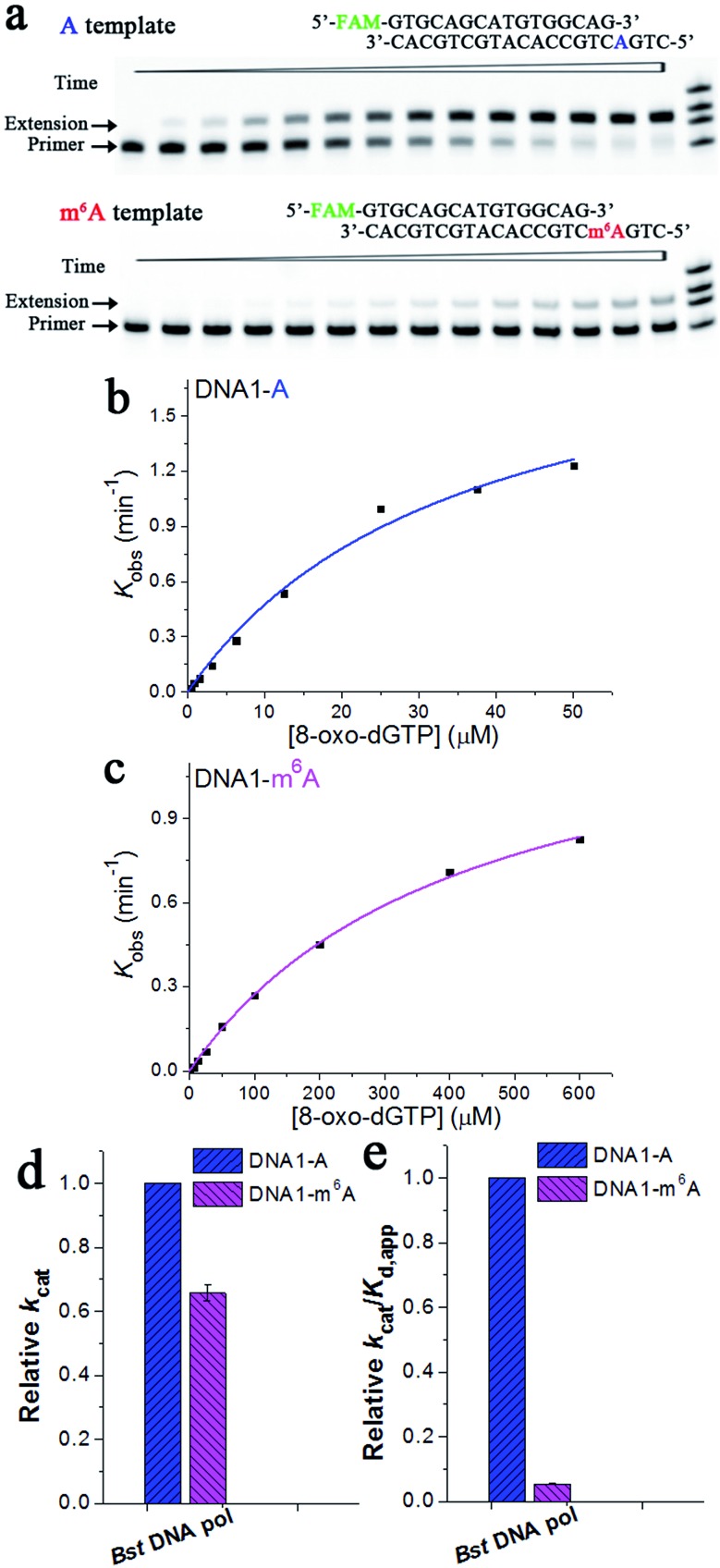
Pre-steady-state kinetics of the 8-oxo-dGTP incorporation by *Bst* DNA pol. (a) Images of representative gels are shown here. Time points are 0, 0.5 min, 1.0 min, 2.0 min, 3.0 min, 4.5 min, 6 min, 8 min, 10 min, 13 min, 16 min, 20 min, 25 min, and 30 min (left to right). Reactions were carried out as described in the ‘Materials and methods’ section using 50 nM primer/template duplex and 3.125 μM 8-oxo-dGTP. The oligonucleotides (primer1, primer1 + 1, primer1 + 2, and primer1 + 3) were used as size markers. (b) and (c) show representative kinetic fitting curves of the 8-oxo-dGTP incorporation opposite m^6^A or A in the DNA template. The 17-nt product was plotted as a function of time and fit to eqn (1) to obtain the reaction rate *k*_obs_. The dependence of *k*_obs_ on the 8-oxo-dGTP concentration was fit to eqn (2) in order to obtain *k*_cat_ and *K*_d,app_. (d) and (e) show the relative kinetic constants (*k*_cat_ or *k*_cat_/*K*_d,app_) of *Bst* DNA pol for the incorporation of 8-oxo-dGTP opposite DNA m^6^A relative to A. All of the kinetic constants are summarized in Table 1.

Extension of the primer by a DNA pol lacking exonuclease activity, such as *Bst* DNA pol, is different from that by those with exonuclease activities. Fig. 7a illustrates representative data with *Bst* DNA pol in the presence of 3.125 μM 8-oxo-dGTP. Specifically, the ‘extension’ band corresponding to the 8-oxo-dGTP incorporation along DNA1-A became clearly evident in about 3 min, while the extension was almost unobservable for DNA1-m^6^A after the same period (Fig. 7a). The results of 8-oxo-dGTP incorporation with *Bst* DNA pol were similar to those obtained with human DNA pol β. Hence, m^6^A methylation in the DNA template leads to evidently compromised incorporation of 8-oxo-dGTP in the complementary strand.

Next, a pre-steady-state single-turnover incorporation assay was performed to obtain quantitative data of *Bst* DNA pol for 8-oxo-dGTP incorporation (Fig. S14 and S15†). Fig. 7b and c illustrate representative kinetic fitting curves of 8-oxo-dGTP incorporation with *Bst* DNA pol opposite m^6^A or A. On the basis of our results, m^6^A methylation in the DNA template leads to a 1.5-fold decrease in *k*_cat_ (Fig. 7d), a 11.3-fold increase in *K*_d,app_, and a 18.8-fold decrease in enzyme efficiency (*k*_cat_/*K*_d,app_, Fig. 7e). These results further evidenced a significant impeding effect of DNA m^6^A on the HG pairing-guided incorporation of 8-oxo-dGTP.

### The m^6^A analysis of synthetic RNA or DNA by 8-oxo-dGTP incorporation

We next seek to explore the potential applications of our findings, for example in determining the m^6^A content of nucleic acids. In this study, various amounts of RNA1-m^6^A were mixed with RNA1-A to mimic samples with diverse m^6^A content (Table S3†). After the 8-oxo-dGTP incorporation step, each sample was analysed using denaturing electrophoresis. The gel image (Fig. S16a†) shows, for a single 8-oxo-dGTP concentration (10 μM), how extended and unextended bands change with m^6^A content from 0 to 100%. Fig. S16b† shows the plot of the fraction of extension *versus* the m^6^A content. These data show an inverse linear relationship, suggesting that our strategy can be used in RNA m^6^A analysis.^15b,27^

We also performed a study to quantitate the DNA m^6^A content. Similarly, various amounts of DNA1-m^6^A were mixed with DNA1-A to mimic samples with diverse methylation levels (Table S4†). Fig. S16c and d† shows the representative gels and the plot of the observed fractions of extension as a function of m^6^A content. The extension percentage is linearly dependent on the methylation level, implying the successful application of our findings in DNA m^6^A analysis.

### Identification of potential m^6^A residues in long RNA

Next, we attempted to test whether our findings can be used for probing m^6^A in long RNA, such as human ribosomal RNA (rRNA). To circumvent the influence of secondary structures on primer hybridization and subsequent extension, we introduced simultaneous control primers (one adjacent to the probed site and the others with known methylation status).^15b,27^ Earlier studies have revealed two well-known m^6^A sites (position 1832 in 18S subunit and 4190 in 28S subunit) and two unmodified A residues (1781 in 18S subunit and 4189 in 28S subunit) in human rRNA.^28^ In this study, fluorescently labeled primers with different lengths were designed such that their 3′ ends lie immediately adjacent to the target residue. The total RNA of cultured HeLa or MCF-7 cells was probed with a primer set (PM1 in Table S5†). As shown in Fig. S17a† (lanes 2 and 6), evident 8-oxo-dGTP incorporation was observed with primer1781 and primer4189, indicating the low methylation level of these sites. By sharp contrast, no 8-oxo-dGTP incorporation was observed with primer1832mA and primer4190mA, indicating the high methylation level of these sites. Our results are consistent with earlier reports by others.^15b,28^

Next, we proceeded to identify potential m^6^A residues in human rRNA. The sequence analysis of the 28S subunit reveals a short fragment between positions 4183 and 4185, which matches the consensus methylation context RAC (R = A or G). However, as such short motifs can be frequently observed in human rRNA, experimental evidence is required. In this study, a different primer set (PM2 in Table S5†) was used. As shown in Fig. S17a† (lanes 4 and 8), more 8-oxo-dGTP was incorporated with primer4183 than with primer4184. Our results suggest that human rRNA is more methylated at position 4184 than position 4183 in the 28S subunit (*P* < 0.05, Fig. S17b†).

## Discussion

8-oxo-dGTP can be formed in the cellular environment by both endogenous oxidation of dGTP and 8-oxo-dG metabolism.^29^ It has been found that innate immune and chemically triggered oxidative stress can modify translational fidelity.^30^ The m^6^A modification represents a naturally occurring and essential modification on nucleic acids and is found within several viruses and most eukaryotes.^31^ Since the discovery of FTO as the first m^6^A demethylase, m^6^A has been found to play important roles in regulating RNA stability, translation, and interactions with other molecules.^32^ An increase in m^6^A at the 5′ UTR of newly transcribed mRNAs in response to heat shock stress has been reported.^33^ Since 8-oxo-dGTP incorporation contributes to mutagenesis and leads to cancer and various heritable diseases,^34^ it is vitally important to reveal the effects of m^6^A methylation on the incorporation of 8-oxo-dGTP into DNA.

Earlier studies demonstrate that human cells may increase the m^6^A levels to get rid of viral infection.^18*b*^ In particular, HIV-1 mRNA contains multiple m^6^A modifications and the infection in CD4^+^ T-cells modifies both host and viral RNAs with m^6^A. Importantly, HIV-1 infections can generate ROS from phagocytes *in vivo*,^35^ thus yielding substantial levels of 8-oxo-dGTP in the deoxynucleotide precursor pool. In the current study, we quantitatively examined the kinetic parameters of a variety of RT enzymes, including the HIV-1 RT, M-MuLV RT, PS RT, and *Bst* DNA pol, using the pre-steady-state single-turnover nucleotide incorporation assay. The incorporation of 8-oxo-dGTP is less likely to occur opposite m^6^A relative to A on RNA templates with all of these enzymes. The data support that the HIV-1 RT was significantly less efficient (13.4-fold discrimination) at incorporating 8-oxo-dGTP opposite m^6^A relative to A. Such a decrease in the catalytic efficiency of HIV-1 RT is mainly caused by the inefficient binding of the m^6^A substrate relative to the A substrate (28.5-fold discrimination). In contrast, the catalytic rate (*k*_cat_) of the HIV-1 RT for the m^6^A substrate is ∼2.1-fold higher than that of the A substrate (Fig. 4c). However, for the M-MuLV RT, PS RT, and *Bst* DNA pol, the m^6^A methylation in the template not only causes a remarkable decrease in substrate binding (*K*_d,app_) but also decreases the catalytic rate. Future structural studies will reveal the molecular details of how the HIV-1 RT accommodates the m^6^A substrate with an increased catalytic rate.

Recently, m^6^A has been reported to be present in eukaryotic genomic DNA.^25^ In the current study, nucleotide incorporation studies have also been carried out with a variety of DNA pol enzymes, including human DNA pol β, φ29 DNA pol, and *Bst* DNA pol. The current study presented the interesting result that human DNA pol β is less efficient at incorporating 8-oxo-dGTP opposite m^6^A relative to opposite A. For *Bst* DNA pol, the pre-steady-state kinetics allows an accurate investigation into the impact of m^6^A methylation on 8-oxo-dGTP incorporation. On the basis of our results, the decrease in catalytic efficiency is collectively caused by the inefficient binding of the m^6^A substrate and the decrease in the catalytic rate (Fig. 7d and e).

8-oxo-dGTP has been found to be present with a high concentration (0.2–2 μM range) in the mitochondrial nucleotide pools of several rat tissues under normal conditions.^8*a*^ Since dGTP is highly susceptible to oxidation, the levels of 8-oxo-dGTP can be substantially increased in the livers of mice subjected to ionizing radiation.^36^ Additionally, the cellular concentration of dTTP is usually about 37 ± 30 μM, whereas tumor cells have concentrations several times higher than those of normal cells.^37^ It has been reported that each copy of HIV-1 genomic RNA contains approximately 3–4 sites with the m^6^A modification,^14*a*^ whereas simian virus 40 mRNA may have more than 10 m^6^A sites.^38^ Indeed, 8-oxo-dGTP can compete with dTTP for incorporation opposite template A to yield A–T to C–G transversions.^8*a*^ Although further evidence is needed, our experimental findings imply that m^6^A methylation may play roles in reducing the mutagenic potential of cellular 8-oxo-dGTP.

The current study, for the first time, demonstrates that a single m^6^A modification is destabilizing to a DNA duplex, possibly because of the relatively unstable base pairing between m^6^A and 8-oxo-dG.^15*a*^ As the incoming nucleotide, the base of 8-oxo-dGTP applies the C_6_ oxygen group (as a hydrogen bond acceptor) and N7 position (as a hydrogen bond donor), which bind the N1 position (as a hydrogen bond acceptor) and the N6 amino group (as a hydrogen bond donor) of the templating A. Earlier studies suggest that the methylamino group of m^6^A in unpaired environments prefers the relaxed (*syn*) orientation,^15*a*^ while the HG pairing requires the flipping of the methylamino group into an energetically unfavorable anti conformation. Because this HG pair (purine–purine) is more bulky than the WC (purine–pyridine) pair, it would be undesirable to incorporate 8-oxo-dGTP opposite m^6^A during DNA synthesis.

In a previous study,^15*b*^ it was reported that a recombinant *Thermus thermophilus* DNA polymerase I (*Tth* pol) expressed a high discrimination for primer extension of T across from A *versus* m^6^A. Now, we explored an application of our discovery and identified a number of enzymes that could potentially provide equivalent discrimination when extending with 8-oxo-dGTP. The success of this approach was illustrated in the current study by detecting methylation levels in human rRNA.

## Conclusions

Most importantly, the current study is the first to disclose that m^6^A methylation significantly impedes the HG pairing-guided incorporation of 8-oxo-dGTP. Although future structural studies of elongating enzymes in complexes with the primer/template containing m^6^A are required in order to demonstrate how m^6^A methylation affects the incorporation, our findings can help advance the understanding of the function of m^6^A in reducing the mutagenic potential of cellular 8-oxo-dGTP. In addition, the impeded incorporation of 8-oxo-dGTP opposite m^6^A was extended to determine m^6^A at a pre-defined position in human rRNA *via* analysis of pausing bands.

## Experimental section

### UV melting studies

UV melting studies were performed using a Jasco-810 spectropolarimeter equipped with a water bath temperature-control accessory. The DNA duplex (10 μM) was incubated in 10 mM Tris–HCl buffer (pH 7.0) containing different concentrations of NaCl. The UV melting profiles were recorded with a heating rate of 0.2 °C min^–1^ and the absorbance values were collected every 1 °C. The melting point (*T*_m_) corresponds to the midtransition temperature, which was determined using the maximum of the first derivative of the absorbance as a function of temperature.

### Pre-steady-state single-turnover 8-oxo-dGTP incorporation assay

This assay was performed according to previous studies.^20^ The 8-oxo-dGTP incorporation scaffold was prepared by incubating the 5′-FAM-labeled primer (DNA) with a template (RNA or DNA) at a molar ratio of 1 : 1.5. The scaffold was then preincubated with a 4-fold excess of the RT or DNA pol enzyme in 1× reaction buffer to make the enzyme:scaffold complex. The reaction was started by rapid mixing of equal volumes of the enzyme:scaffold complex with a solution containing two-fold concentrations of 8-oxo-dGTP in 1× reaction buffer. Reactions were stopped at various times by the addition of a 4.5-fold excess of quenching solution (95% formamide, 25 mM EDTA at pH 8.0). Products were separated by electrophoresis on a standard polyacrylamide denaturing gel (19 : 1, 20%) and scanned using a phosphorimager. The percentage of primer extended was quantified, and plotted *vs.* time for each concentration of 8-oxo-dGTP used. The recipe of each reaction buffer and the final concentrations of scaffold and enzyme are provided in the ESI.†

### Data analysis

The data were analyzed by fitting the curve using nonlinear regression with ORIGIN 8.5 software (OriginLab Corporation, Massachusetts, USA). The time courses of primer extension reactions were fit to eqn (1). The observed rates (*k*_obs_) thus obtained were further plotted as a function of the substrate concentration and then fit to eqn (2) in order to obtain values for the maximum rate of 8-oxo-dGTP incorporation (*k*_cat_) and apparent *K*_d_ (*K*_d,app_) governing 8-oxo-dGTP binding.^20^

1 Product = *a* × e^–*k*_obs_*t*^ + *b*

2 *k*_obs_ = *k*_cat_ × [substrate]/(*K*_d,app_ + [substrate])

Discrimination was calculated using the ratio of the specificity constants for the m^6^A template over the A template.

### Identification of potential m^6^A residue in human rRNA

For each 10 μL reaction, 2.0 μg total RNA and each primer at 40 nM were used. The reaction was performed with an incubation temperature of 45 °C for 30 min in 1× ThermoPol™ buffer, in the presence of 1.0 U *Bst* DNA polymerase and 10 μM 8-oxo-dGTP. Full experimental details are described in ESI.†

### Statistical analysis

Statistical analysis was performed using ORIGIN 8.5 software. The methylation differences were considered to be significant for *P* < 0.05.
