# Supplementary material for: The m6A methylation perturbs the Hoogsteen pairing-guided incorporation of an oxidized nucleotide
Source: Chem Sci. 2017 Jul 6;8(9):6380–8. doi: 10.1039/c7sc02340e (PMC5628602; doi:10.1039/c7sc02340e)
Supplement: Supplementary file 1 [file SC-008-C7SC02340E-s001.pdf]

Electronic Supplementary Material (ESI) for Chemical Science.
This journal is © The Royal Society of Chemistry 2017

Supporting Information

The m⁶A methylation perturbs Hoogsteen pairing-guided incorporation of an oxidized nucleotide

Shaoru Wang, Yanyan Song, Yafen Wang, Xin Li, Boshi Fu, Yinong Liu, Jiaqi Wang, Lai Wei, Tian Tian*, Xiang Zhou*

Email: xzhou@whu.edu.cn, ttian@whu.edu.cn

Table of contents

Table S1	Sequences of oligomers used in 8-oxo-dGTP incorporation study
Table S2	The sequences of duplexes used in UV melting studies
Table S3	Samples used in RNA methylation analysis
Table S4	Samples used in DNA methylation analysis
Table S5	Primers used in m^{6}A analysis of human rRNA
Figure S1	Chemical structures of the Watson-Crick (WC) base pairs and Hoogsteen (HG) base pairs
Figure S2	The m^{6}A methylation destabilizes HG base pairs in paired region of DNA duplexes
Figure S3	Impeded 8-oxo-dGTP incorporation by HIV-1 RT opposite m^{6}A relative to A
Figure S4	Pre-steady-state kinetics of 8-oxo-dGTP incorporation opposite RNA A by HIV-1 RT
Figure S5	Pre-steady-state kinetics of 8-oxo-dGTP incorporation opposite RNA m^{6}A by HIV-1 RT
Figure S6	Pre-steady-state kinetics of 8-oxo-dGTP incorporation opposite RNA A by M-MuLV RT
Figure S7	Pre-steady-state kinetics of 8-oxo-dGTP incorporation opposite RNA m^{6}A by M-MuLV RT
Figure S8	Pre-steady-state kinetics of 8-oxo-dGTP incorporation opposite RNA A by PS RT
Figure S9	Pre-steady-state kinetics of 8-oxo-dGTP incorporation opposite RNA m^{6}A by PS RT
Figure S10	Pre-steady-state kinetics of 8-oxo-dGTP incorporation opposite RNA A by *Bst* DNA pol
Figure S11	Pre-steady-state kinetics of 8-oxo-dGTP incorporation opposite RNA m^{6}A by *Bst* DNA pol

Figure S12	Impeded 8-oxo-dGTP incorporation by human DNA pol β opposite m^{6}A relative to A
Figure S13	Impeded 8-oxo-dGTP incorporation by human DNA pol β opposite m^{6}A relative to A
Figure S14	Pre-steady-state kinetics of 8-oxo-dGTP incorporation opposite DNA A by *Bst* DNA pol
Figure S15	Pre-steady-state kinetics of 8-oxo-dGTP incorporation opposite DNA m^{6}A by *Bst* DNA pol
Figure S16	Quantitative analysis of the m^{6}A content of RNA or DNA samples
Figure S17	The m^{6}A analysis of the 28S rRNA of cultured human cells

Materials

HIV-1 Reverse Transcriptase, Recombinant, *E. coli* (product number: 382129) was purchased from EMD Millipore Corporation (Merck KGaA, Germany). Human DNA polymerase β (product number. 1077) was purchased from CHIMERx (Madison, WI, USA). Moloney Murine Leukemia Virus (M-MuLV) Reverse Transcriptase (product number: M0253), ProtoScript® II Reverse Transcriptase (product number: M0368), *Bst* DNA polymerase, Large Fragment (product number: M0275) and Bacteriophage ϕ29 DNA polymerase (product number: M0269) were purchased from New England Biolabs, Inc. (UK). 8-Oxo-2'-deoxyguanosine-5'-Triphosphate (8-Oxo-dGTP) was purchased from Trilink Biotechnologies Inc. (San Diego, CA, USA). All oligonucleotides were purchased from Takara Corporation (Dalian, China). Fetal Bovine Serum (FBS) was purchased from HyClone Inc. DMEM medium and Pen Strep antibiotic were purchased from Gibco, Life Technologies. TRIzol was purchased from Invitrogen Inc. RNeasy Mini Kit was purchased from Qiagen Inc. The chemical reagents were purchased from Sigma Inc. Polyacrylamide gel were scanned with Pharos FX Molecular imager (Bio-Rad, USA) operated in the fluorescence mode. The Discovery Series Quantity One 1-D Analysis Software Version 4.6.9 was used to determine the percentage of extended primers. Each band was enclosed in a box of the same area, and the volume calculated. Background was manually subtracted.

Methods

The incorporation of 8-oxo-dGTP by HIV-1 Reverse Transcriptase This reaction was performed in 1 × HIV-1 Reverse Transcriptase reaction buffer, which contained 50 mM Tris-HCl (pH 8.3), 6 mM $MgCl_2$ and 40 mM KCl. The reaction was started by rapid mixing of equal volumes of the enzyme:scaffold complex with an solution containing twofold concentrations of 8-oxo-dGTP in 1 × reaction buffer. The final concentration of each component for a typical 10 μL reaction are as follow: 200 nM HIV-1 Reverse Transcriptase, 50 nM 5'-FAM-labeled primer (DNA), 75 nM template (RNA) and various 8-oxo-dGTP concentrations. Incubation was carried out at 37 °C. The reactions were quenched at various times by adding 45 μL stop buffer (95% formamide, 25 mM EDTA at pH 8.0), followed by immediate heating at 90 °C for 10 min. After cooling down to 4 °C, products were separated by electrophoresis from the unextended primer on a standard polyacrylamide denaturing gel (20%, 19:1) and scanned using a phosphorimager.

The incorporation of 8-oxo-dGTP by M-MuLV Reverse Transcriptase This reaction was performed in 1 × M-MuLV Reverse Transcriptase reaction buffer, which contained 50 mM Tris-HCl, 75 mM KCl, 3 mM $MgCl_2$, 10 mM DTT, pH 8.3 @ 25°C. The reaction was started by rapid mixing of equal volumes of the enzyme:scaffold complex with an solution containing twofold concentrations of 8-oxo-dGTP in 1 × reaction buffer. The final concentration of each component for a typical 10 μL reaction are as follow: 200 nM M-MuLV Reverse Transcriptase, 50 nM 5'-FAM-labeled primer (DNA), 75 nM template (RNA) and various 8-oxo-dGTP concentrations. Incubation was carried out at 42 °C. The reactions were quenched at various times by adding 45

μL stop buffer (95% formamide, 25 mM EDTA at pH 8.0), followed by immediate heating at 90 °C for 10 min. After cooling down to 4 °C, products were separated by electrophoresis from the unextended primer on a standard polyacrylamide denaturing gel (20%, 19:1) and scanned using a phosphorimager.

The incorporation of 8-oxo-dGTP by ProtoScript® II Reverse Transcriptase This reaction was performed in 1 × ProtoScript® II Reverse Transcriptase reaction buffer, which contained 50 mM Tris-HCl, 75 mM KCl, 3 mM $MgCl_2$, pH 8.3 @ 25°C. The reaction was started by rapid mixing of equal volumes of the enzyme:scaffold complex with an solution containing twofold concentrations of 8-oxo-dGTP in 1 × reaction buffer. The final concentration of each component for a typical 10 μL reaction are as follow: 200 nM ProtoScript® II Reverse Transcriptase, 50 nM 5'-FAM-labeled primer (DNA), 75 nM template (RNA) and various 8-oxo-dGTP concentrations. Incubation was carried out at 42 °C. The reactions were quenched at various times by adding 45 μL stop buffer (95% formamide, 25 mM EDTA at pH 8.0), followed by immediate heating at 90 °C for 10 min. After cooling down to 4 °C, products were separated by electrophoresis from the unextended primer on a standard polyacrylamide denaturing gel (20%, 19:1) and scanned using a phosphorimager.

The incorporation of 8-oxo-dGTP by *Bst* DNA polymerase This reaction was performed in 1 × ThermoPol™ reaction buffer, which contained 20 mM Tris-HCl, 10 mM $(NH_4)_2SO_4$, 10 mM KCl, 2 mM $MgSO_4$ and 0.1 % Triton X-100 at pH 8.8 @ 25 °C. The reaction was started by rapid mixing of equal volumes of the enzyme:scaffold complex with an solution containing twofold concentrations of 8-oxo-dGTP in 1 × reaction buffer. The final concentration of each component for a typical 10 μL reaction are as follow: 200 nM *Bst* DNA polymerase, 50 nM 5'-FAM-labeled primer (DNA), 75 nM template (RNA or DNA) and various 8-oxo-dGTP concentrations. Incubation was carried out at 45 °C. The reactions were quenched at various times by adding 45 μL stop buffer (95% formamide, 25 mM EDTA at pH 8.0), followed by immediate heating at 90 °C for 10 min. After cooling down to 4 °C, products were separated by electrophoresis from the unextended primer on a standard polyacrylamide denaturing gel (20%, 19:1) and scanned using a phosphorimager.

The incorporation of 8-oxo-dGTP by human DNA polymerase β This reaction was performed in 1 × human DNA polymerase β reaction buffer, which contained 50 mM Tris-HCl (pH 8.7), 100 mM KCl, 10 mM $MgCl_2$, 0.4 mg/ml bovine serum albumin, 1.0 mM dithiothreitol, 15% (v/v) glycerol. The reaction was started by rapid mixing of equal volumes of the enzyme:scaffold complex with an solution containing twofold concentrations of 8-oxo-dGTP in 1 × reaction buffer. The final concentration of each component for a typical 10 μL reaction are as follow: 0.1 U human DNA polymerase β, 50 nM 5'-FAM-labeled primer (DNA), 75 nM template (DNA) and various 8-oxo-dGTP concentrations. Incubation was carried out at 37 °C. The reactions were quenched at various times by adding 45 μL stop buffer (95% formamide, 25 mM EDTA at pH 8.0), followed

by immediate heating at 90 °C for 10 min. After cooling down to 4 °C, products were separated by electrophoresis from the unextended primer on a standard polyacrylamide denaturing gel (20%, 19:1) and scanned using a phosphorimager.

The incorporation of 8-oxo-dGTP by Bacteriophage ϕ29 DNA polymerase This reaction was performed in 1 × Bacteriophage ϕ29 DNA polymerase reaction buffer, which contained 50 mM Tris-HCl, 10 mM $MgCl_2$, 10 mM $(NH_4)_2SO_4$, 4 mM DTT, pH 7.5 @ 25°C. The reaction was started by rapid mixing of equal volumes of the enzyme:scaffold complex with an solution containing twofold concentrations of 8-oxo-dGTP in 1 × reaction buffer. The final concentration of each component for a typical 10 μL reaction are as follow: 10 U ϕ29 DNA polymerase, 50 nM 5'-FAM-labeled primer (DNA), 75 nM template (DNA) and various 8-oxo-dGTP concentrations. Incubation was carried out at 30 °C. The reactions were quenched at various times by adding 45 μL stop buffer (95% formamide, 25 mM EDTA at pH 8.0), followed by immediate heating at 90 °C for 10 min. After cooling down to 4 °C, products were separated by electrophoresis from the unextended primer on a standard polyacrylamide denaturing gel (20%, 19:1) and scanned using a phosphorimager.

Isolation of total RNA from cultured tumor cells HeLa cells and MCF-7 cells were obtained from CCTCC and grown in DMEM medium containing 10% Fetal Bovine Serum (FBS) and 1% Pen Strep at 37 °C, 5% CO_2 humidified atmosphere. A standard Trizol extraction was performed, and the aqueous phase was mixed with 1 volume of RNAse free ethanol, followed by the use of RNeasy Mini Kit. The following purification was performed according to the protocol by manufacturer. The RNA sample was quantitated on a NanoDrop 2000c (Thermo Scientific).

The m^6A analysis of synthetic RNA or DNA by 8-oxo-dGTP incorporation Each reaction was performed in 1 × ThermoPol™ reaction buffer in the presence of 1.0 U *Bst* DNA polymerase, 10 μM 8-oxo-dGTP, 40 nM primer1, 50 nM sample (RNA or DNA). After incubation at 45 °C for 30 min, each reaction (10 μL) was quenched by adding 45 μL stop buffer (95% formamide, 25 mM EDTA at pH 8.0). The solutions were immediately heated at 90 °C for 10 min. After cooling down to 4 °C, products were separated by electrophoresis from the unextended primer on a standard polyacrylamide denaturing gel (20%) and scanned using a phosphorimager.

Identification of potential m^6A residue in human rRNA For each reaction, 2.0 μg total RNA and each primer at 40 nM were used. Each reaction was performed in 1 × ThermoPol™ reaction buffer in the presence of 1.0 U *Bst* DNA polymerase, 10 μM 8-oxo-dGTP. After incubation at 45 °C for 30 min, each reaction (10 μL) was quenched by adding 45 μL stop buffer (95% formamide, 25 mM EDTA at pH 8.0). The solutions were immediately heated at 90 °C for 10 min. After cooling down to 4 °C, products were separated by electrophoresis from the unextended primer on a standard polyacrylamide denaturing gel (20%) and scanned using a phosphorimager.

Table S1. Sequences of oligomers used in 8-oxo-dGTP incorporation study

Oligomer	Sequence(from 5’to 3’)	Scaffold
m^{6}A sequence	5’-CTGACT-**m^{6}A**-ATGCTG-3’	
OG sequence	5’-CAGCAT-**8-oxo-dG**-AGTCAG-3’	
A sequence	5’-CTGACT-**A**-ATGCTG-3’	
T sequence	5’-CAGCAT-**T**-AGTCAG-3’	
C sequence	5’-CTGACT-**C**-ATGCTG-3’	
G sequence	5’-CAGCAT-**G**-AGTCAG-3’	
RNA1-A	5’-AG**A**CUGCCACAUGCUGCAC-3’	Scaffold 1
RNA1-m^{6}A	5’-AG-**m^{6}A**-CUGCCACAUGCUGCAC-3’	
primer1	5’-FAM-GTGCAGCATGTGGCAG-3’	
RNA2-A	5’-AG**A**GUGCCACAUGCUGCAC-3’	Scaffold 2
RNA2-m^{6}A	5’-AG-**m^{6}A**-GUGCCACAUGCUGCAC-3’	
primer2	5’-FAM-GTGCAGCATGTGGCAC-3’	
RNA3-A	5’-AG**A**AUGCCACAUGCUGCAC-3’	Scaffold 3
RNA3-m^{6}A	5’-AG-**m^{6}A**-AUGCCACAUGCUGCAC-3’	
primer3	5’-FAM-GTGCAGCATGTGGCAT-3’	
RNA4-A	5’-AG**A**UUGCCACAUGCUGCAC-3’	Scaffold 4
RNA4-m^{6}A	5’-AG-**m^{6}A**-UUGCCACAUGCUGCAC-3’	
primer4	5’-FAM-GTGCAGCATGTGGCAA-3’	
DNA1-A	5’-CTG**A**CTGCCACATGCTGCAC-3’	Scaffold 1 (primer1)
DNA1-m^{6}A	5’-CTG-**m^{6}A**-CTGCCACATGCTGCAC-3’	
DNA2-A	5’-CTG**A**GTGCCACATGCTGCAC-3’	Scaffold 2 (primer2)
DNA2-m^{6}A	5’-CTG-**m^{6}A**-GTGCCACATGCTGCAC-3’	
DNA3-A	5’-CTG**A**ATGCCACATGCTGCAC-3’	Scaffold 3 (primer3)
DNA3-m^{6}A	5’-CTG-**m^{6}A**-ATGCCACATGCTGCAC-3’	
DNA4-A	5’-CTG**A**TTGCCACATGCTGCAC-3’	Scaffold 4 (primer4)
DNA4-m^{6}A	5’-CTG-**m^{6}A**-TTGCCACATGCTGCAC-3’	
primer1+1	5’-FAM-GTGCAGCATGTGGCAGG-3’	
primer1+2	5’-FAM-GTGCAGCATGTGGCAGGC-3’	
primer1+3	5’-FAM-GTGCAGCATGTGGCAGGCT-3’	
primer1781A	5’-FAM-CCTCTAGATAGTCAAGTTCGACCGTCTTCTCAGCGC-3’	
primer1832mA	5’-FAM-TCCTTCCGCAGGTTCACCTACGGAAACCTTG-3’	
primer4189A	5’-FAM-CCTGAGCTCGCCTTAGGACACCTGCGT-3’	
primer4190mA	5’-FAM-GAGCTCGCCTTAGGACACCTGCG-3’	
primer4183	5’-FAM-CTCGCCTTAGGACACCTGCGTTACCGT-3’	
primer4184	5’-FAM-GCCTTAGGACACCTGCGTTACCG-3’	

Table S2. The sequences of duplexes used in UV melting studies

Name	Sequence
**m⁶A:OG**	5'-CTGACT **m⁶A** ATGCTG-3'<br>3'-GACTGA **8-oxo-dG** TACGAC-5'
**A:OG**	5'-CTGACT **A** ATGCTG-3'<br>3'-GACTGA **8-oxo-dG** TACGAC-5'
**A:T**	5'-CTGACT **A** ATGCTG-3'<br>3'-GACTGA **T** TACGAC-5'
**C:G**	5'-CTGACT **C** ATGCTG-3'<br>3'-GACTGA **G** TACGAC-5'

Table S3. Samples used in RNA methylation analysis

Sample	Components (Final Concentration)	m⁶A content (%)
RNA-m1	RNA1-A (500 nM)	0
RNA-m2	RNA1-A (450 nM) and RNA1-m⁶A (50 nM)	10
RNA-m3	RNA1-A (400 nM) and RNA1-m⁶A (100 nM)	20
RNA-m4	RNA1-A (350 nM) and RNA1-m⁶A (150 nM)	30
RNA-m5	RNA1-A (300 nM) and RNA1-m⁶A (200 nM)	40
RNA-m6	RNA1-A (250 nM) and RNA1-m⁶A (250 nM)	50
RNA-m7	RNA1-A (200 nM) and RNA1-m⁶A (300 nM)	60
RNA-m8	RNA1-A (150 nM) and RNA1-m⁶A (350 nM)	70
RNA-m9	RNA1-A (100 nM) and RNA1-m⁶A (400 nM)	80
RNA-m10	RNA1-A (50 nM) and RNA1-m⁶A (450 nM)	90
RNA-m11	RNA1-m⁶A (500 nM)	100

Table S4. Samples used in DNA methylation analysis

Sample	Components (Final Concentration)	m⁶A content (%)
DNA-m1	DNA1-m⁶A (500 nM)	100
DNA-m2	DNA1-m⁶A (450 nM) and DNA-A (50 nM)	90
DNA-m3	DNA1-m⁶A (400 nM) and DNA-A (100 nM)	80
DNA-m4	DNA1-m⁶A (350 nM) and DNA-A (150 nM)	70
DNA-m5	DNA1-m⁶A (300 nM) and DNA-A (200 nM)	60
DNA-m6	DNA1-m⁶A (250 nM) and DNA-A (250 nM)	50
DNA-m7	DNA1-m⁶A (200 nM) and DNA-A (300 nM)	40
DNA-m8	DNA1-m⁶A (150 nM) and DNA-A (350 nM)	30
DNA-m9	DNA1-m⁶A (100 nM) and DNA-A (400 nM)	20
DNA-m10	DNA1-m⁶A (50 nM) and DNA-A (450 nM)	10
DNA-m11	DNA-A (500 nM)	0

Table S5. Primers used in m⁶A analysis of human rRNA

Primer sets	Components			
PM1	primer1781A	primer1832mA	primer4189A	primer4190mA
PM2	primer1781A	primer1832mA	primer4184	primer4183

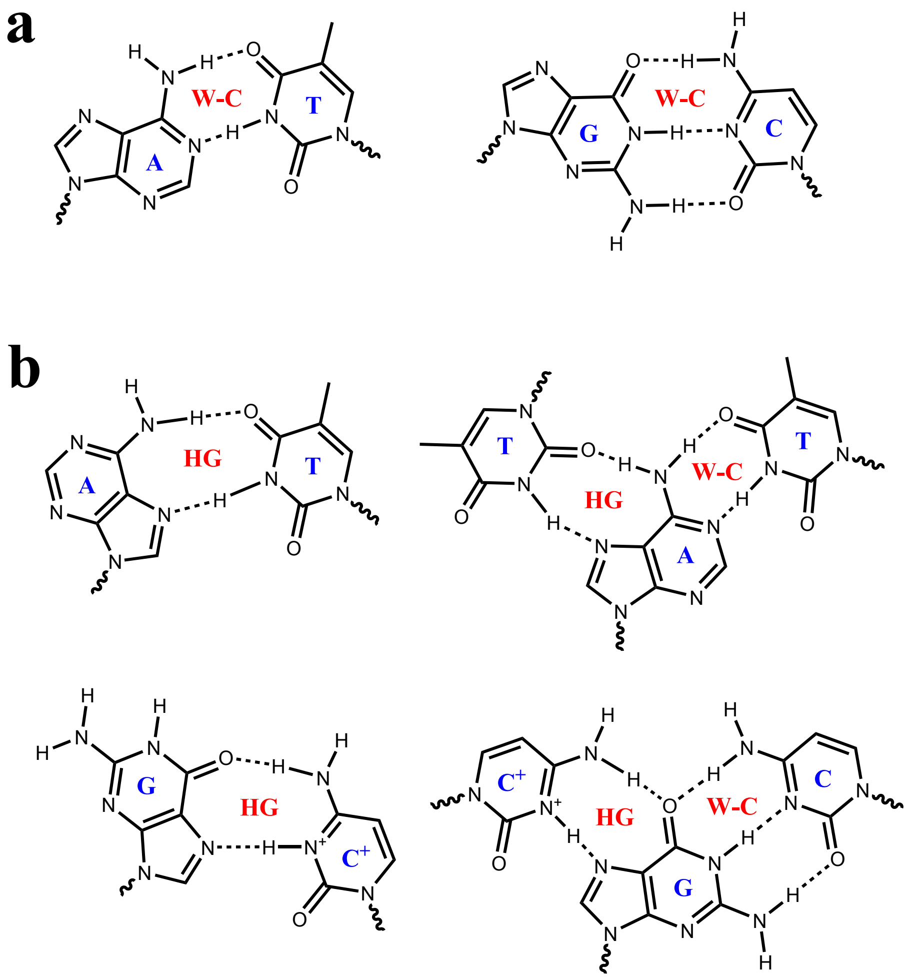

Figure S1. Chemical structures of the Watson-Crick (WC) base pairs and Hoogsteen (HG) base pairs.

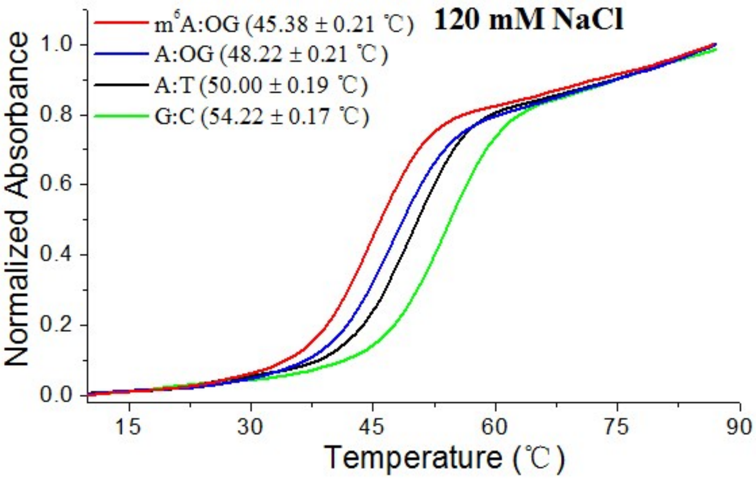

Figure S2. The m^6A methylation destabilizes HG base pairs in paired region of DNA duplexes. Representative melting profiles of different duplexes (10 μM) were recorded in 10 mM Tris-HCl buffer (pH 7.0, 120 mM NaCl).

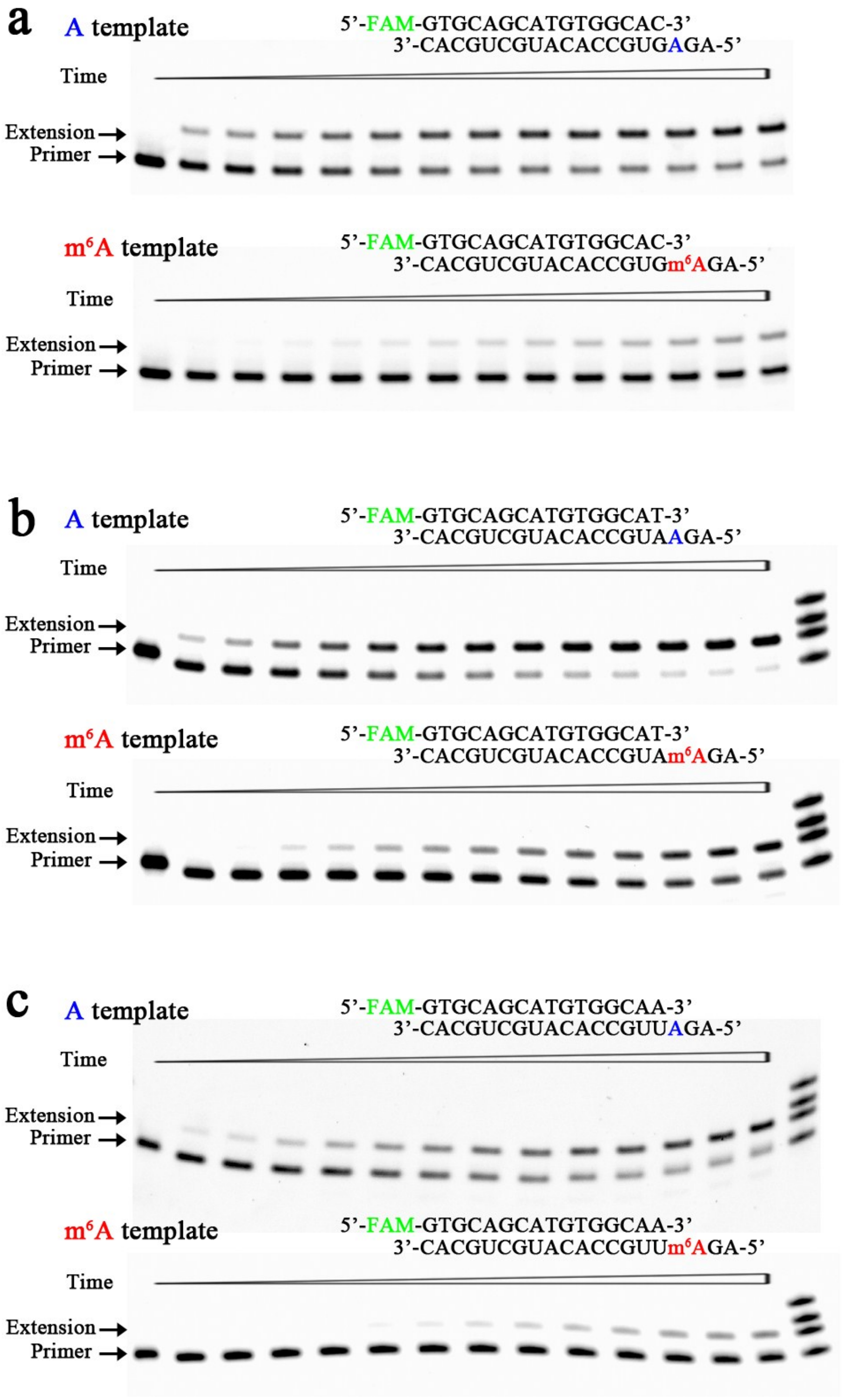

Figure S3. Impeded 8-oxo-dGTP incorporation by HIV-1 RT opposite m^{6}A relative to A. Image of representative gels is shown here. Reactions were carried out as described in 'Materials and Methods' section using 50 nM primer/template duplex and 98 nM 8-oxo-dGTP (**a**, scaffold 2; **b**, scaffold 3; **c**, scaffold 4) for various reaction times. Time points are 0, 0.5 min, 1.0 min, 2.0 min, 3.0 min, 4.5 min, 6 min, 8 min, 10 min, 13 min, 16 min, 20 min, 25 min and 30 min (left to right). The oligonucleotides (primer1, primer1+1, primer1+2 and primer1+3) were used as size markers in various gels.

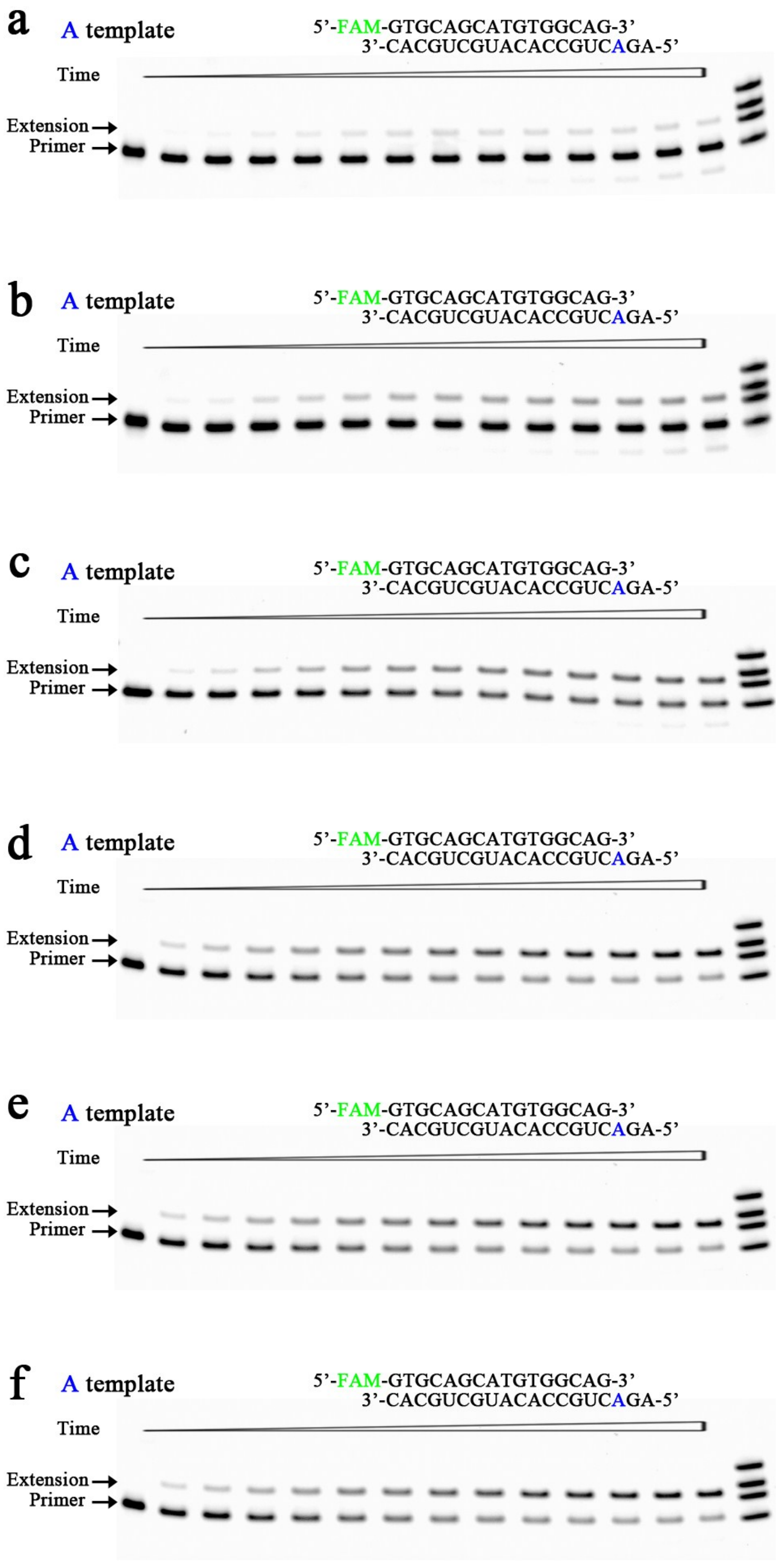
a
A template
5'-FAM-GTGCAGCATGTGGCAG-3'
3'-CACGUCGUACACCGUCAGA-5'
Time
Extension
Primer
b
A template
5'-FAM-GTGCAGCATGTGGCAG-3'
3'-CACGUCGUACACCGUCAGA-5'
Time
Extension
Primer
c
A template
5'-FAM-GTGCAGCATGTGGCAG-3'
3'-CACGUCGUACACCGUCAGA-5'
Time
Extension
Primer
d
A template
5'-FAM-GTGCAGCATGTGGCAG-3'
3'-CACGUCGUACACCGUCAGA-5'
Time
Extension
Primer
e
A template
5'-FAM-GTGCAGCATGTGGCAG-3'
3'-CACGUCGUACACCGUCAGA-5'
Time
Extension
Primer
f
A template
5'-FAM-GTGCAGCATGTGGCAG-3'
3'-CACGUCGUACACCGUCAGA-5'
Time
Extension
Primer

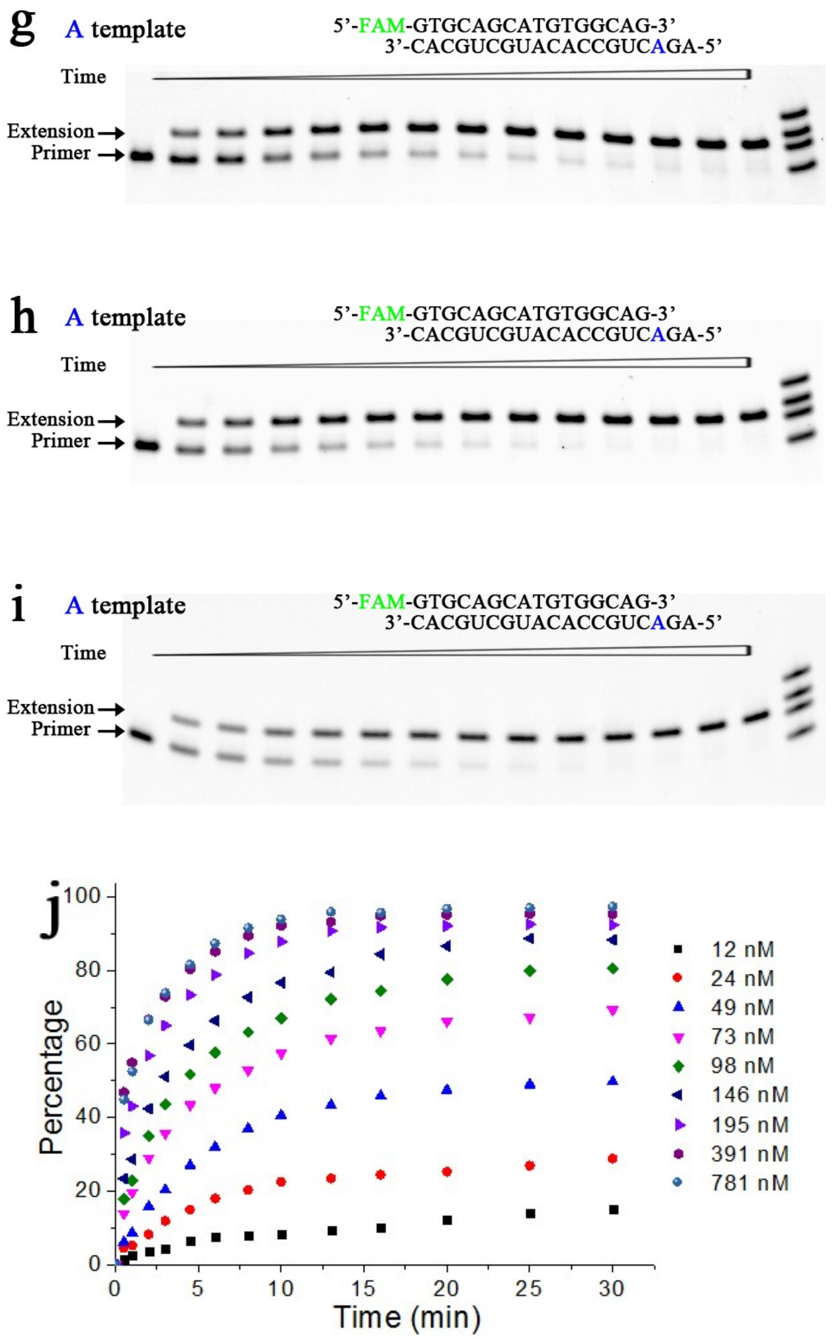

Figure S4. Pre-steady-state kinetics of 8-oxo-dGTP incorporation opposite RNA A by HIV-1 RT. Image of representative gels is shown here. Reactions were carried out as described in ‘Materials and Methods’ section. **a**-**i**, Preincubated HIV-1 RT and 50 nM primer/template duplex were mixed with various concentrations of 8-oxo-dGTP (**a**, 12 nM; **b**, 24 nM; **c**, 49 nM; **d**, 73 nM; **e**, 98 nM; **f**, 146 nM; **g**, 195 nM; **h**, 391 nM; **i**, 781 nM) for various reaction times. Time points are 0, 0.5 min, 1.0 min, 2.0 min, 3.0 min, 4.5 min, 6 min, 8 min, 10 min, 13 min, 16 min, 20 min, 25 min and 30 min (left to right). The oligonucleotides (primer1, primer1+1, primer1+2 and primer1+3) were used as size markers in various gels. **j**. Kinetic curves of 8-oxo-dGTP incorporation opposite A.

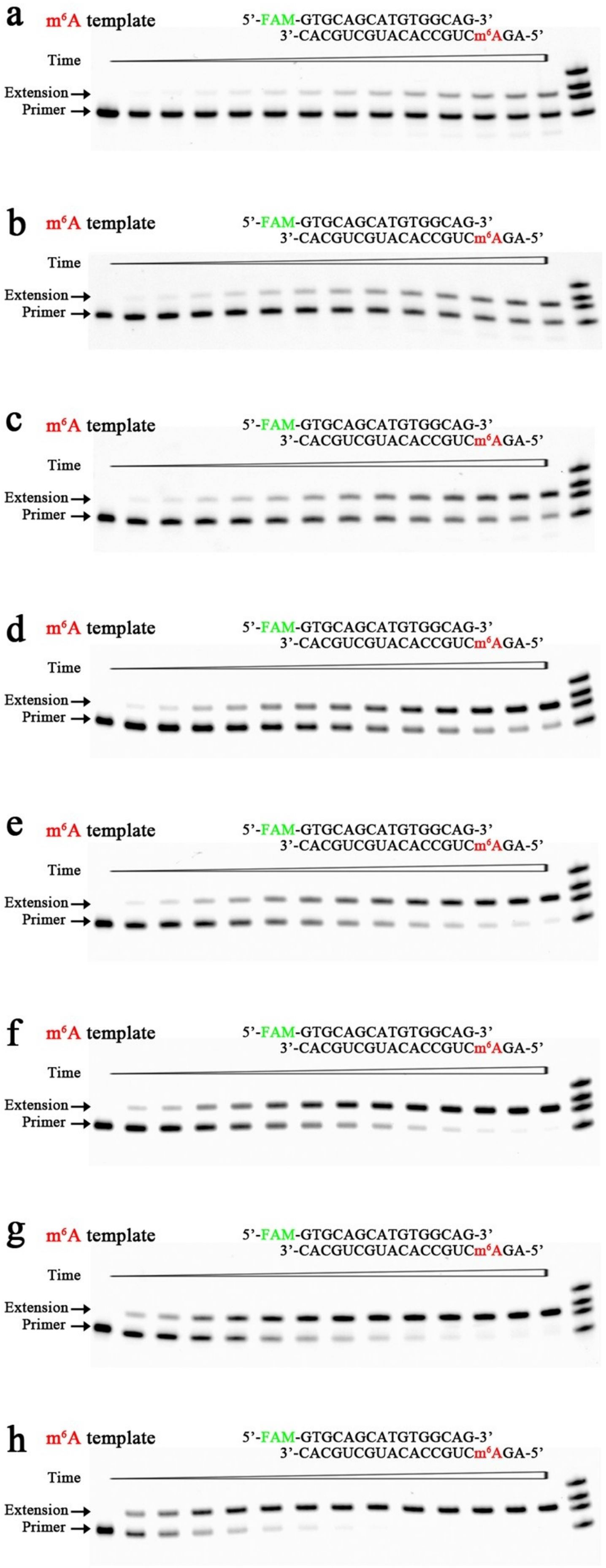
a
m6A template
5'-FAM-GTGCAGCATGTGGCAG-3'
3'-CACGUCGUACACCGUCm6AGA-5'
Time
Extension
Primer
b
m6A template
5'-FAM-GTGCAGCATGTGGCAG-3'
3'-CACGUCGUACACCGUCm6AGA-5'
Time
Extension
Primer
c
m6A template
5'-FAM-GTGCAGCATGTGGCAG-3'
3'-CACGUCGUACACCGUCm6AGA-5'
Time
Extension
Primer
d
m6A template
5'-FAM-GTGCAGCATGTGGCAG-3'
3'-CACGUCGUACACCGUCm6AGA-5'
Time
Extension
Primer
e
m6A template
5'-FAM-GTGCAGCATGTGGCAG-3'
3'-CACGUCGUACACCGUCm6AGA-5'
Time
Extension
Primer
f
m6A template
5'-FAM-GTGCAGCATGTGGCAG-3'
3'-CACGUCGUACACCGUCm6AGA-5'
Time
Extension
Primer
g
m6A template
5'-FAM-GTGCAGCATGTGGCAG-3'
3'-CACGUCGUACACCGUCm6AGA-5'
Time
Extension
Primer
h
m6A template
5'-FAM-GTGCAGCATGTGGCAG-3'
3'-CACGUCGUACACCGUCm6AGA-5'
Time
Extension
Primer

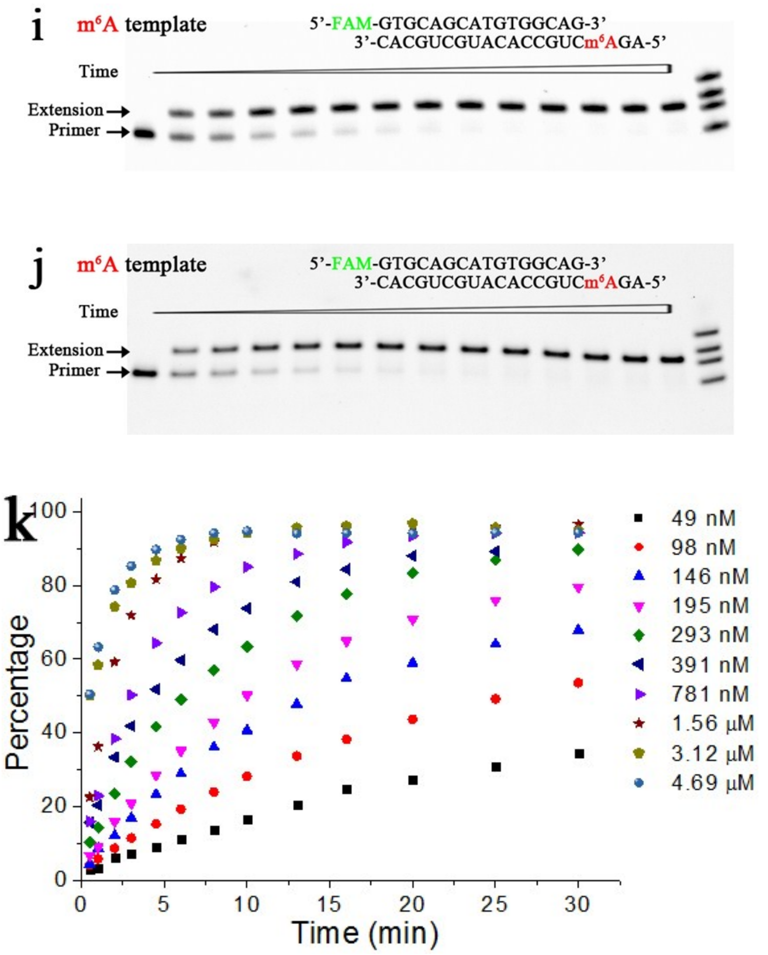

Figure S5. Pre-steady-state kinetics of 8-oxo-dGTP incorporation opposite RNA m^{6}A by HIV-1 RT. Image of representative gels is shown here. Reactions were carried out as described in 'Materials and Methods' section. **a**-**j**, Preincubated HIV-1 RT and 50 nM primer/template duplex were mixed with various concentrations of 8-oxo-dGTP (**a**, 49 nM; **b**, 98 nM; **c**, 146 nM; **d**, 195 nM;□**e**, 293 nM; **f**, 391 nM; **g**, 781 nM; **h**, 1.56 µM; **i**, 3.12 µM; **j**, 4.69 µM) for various reaction times. Time points are 0, 0.5 min, 1.0 min, 2.0 min, 3.0 min, 4.5 min, 6 min, 8 min, 10 min, 13 min, 16 min, 20 min, 25 min and 30 min (left to right). The oligonucleotides (primer1, primer1+1, primer1+2 and primer1+3) were used as size markers in various gels. **k**. Kinetic curves of 8-oxo-dGTP incorporation opposite m^{6}A.

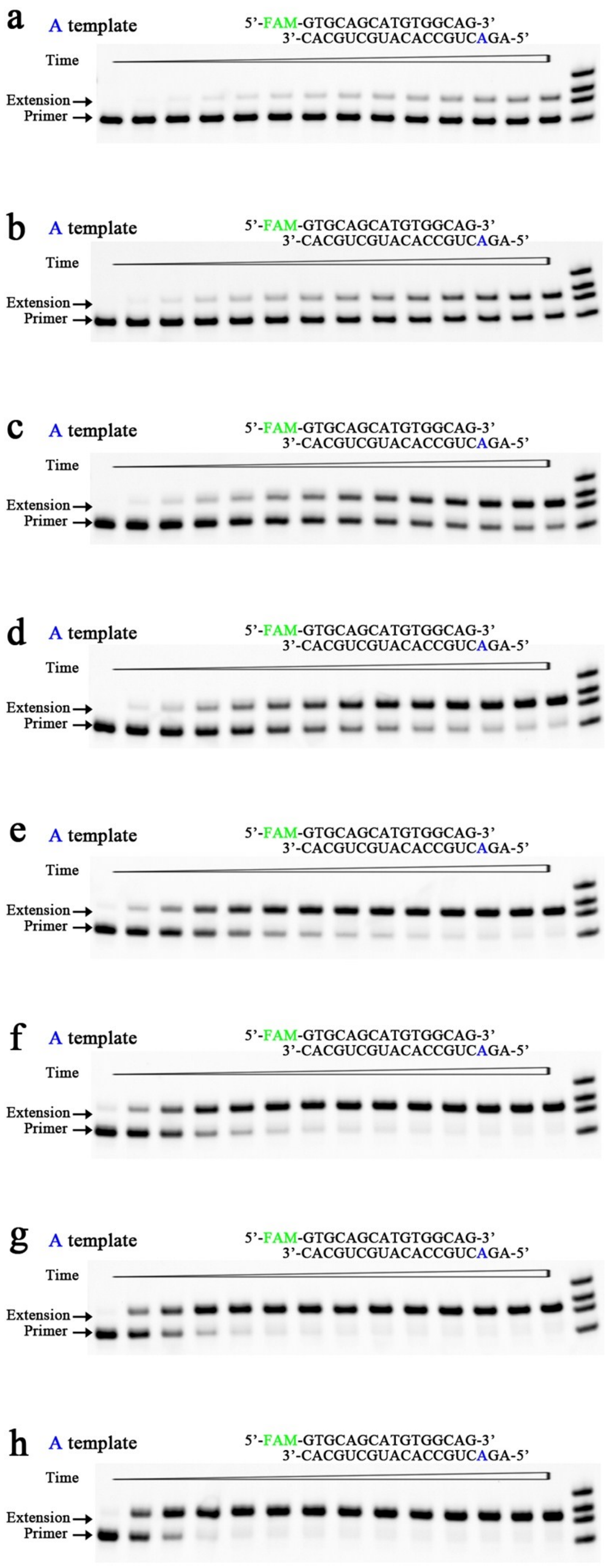
a
A template
5’-FAM-GTGCAGCATGTGGCAG-3’
3’-CACGUCGUACACCGUCAGA-5’
Time
Extension
Primer
b
A template
5’-FAM-GTGCAGCATGTGGCAG-3’
3’-CACGUCGUACACCGUCAGA-5’
Time
Extension
Primer
c
A template
5’-FAM-GTGCAGCATGTGGCAG-3’
3’-CACGUCGUACACCGUCAGA-5’
Time
Extension
Primer
d
A template
5’-FAM-GTGCAGCATGTGGCAG-3’
3’-CACGUCGUACACCGUCAGA-5’
Time
Extension
Primer
e
A template
5’-FAM-GTGCAGCATGTGGCAG-3’
3’-CACGUCGUACACCGUCAGA-5’
Time
Extension
Primer
f
A template
5’-FAM-GTGCAGCATGTGGCAG-3’
3’-CACGUCGUACACCGUCAGA-5’
Time
Extension
Primer
g
A template
5’-FAM-GTGCAGCATGTGGCAG-3’
3’-CACGUCGUACACCGUCAGA-5’
Time
Extension
Primer
h
A template
5’-FAM-GTGCAGCATGTGGCAG-3’
3’-CACGUCGUACACCGUCAGA-5’
Time
Extension
Primer

i

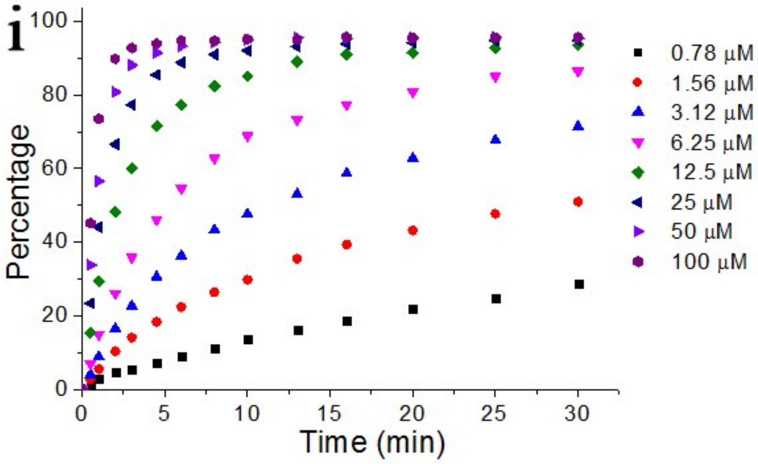

j

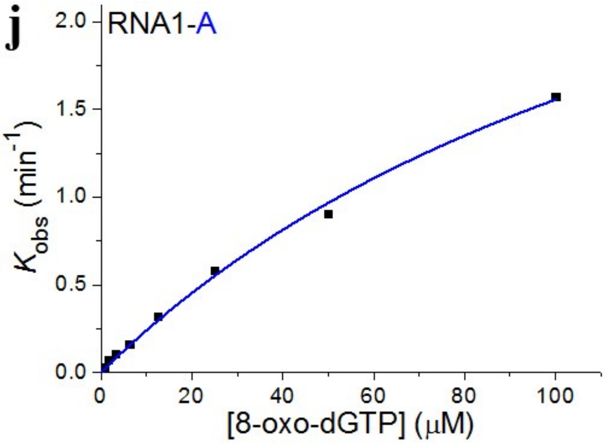

Figure S6. Pre-steady-state kinetics of 8-oxo-dGTP incorporation opposite RNA A by M-MuLV RT. Image of representative gels is shown here. Reactions were carried out as described in 'Materials and Methods' section. **a-h**, Preincubated M-MuLV RT and 50 nM primer/template duplex were mixed with various concentrations of 8-oxo-dGTP (**a**, 0.78 μM; **b**, 1.56 μM; **c**, 3.12 μM; **d**, 6.25 μM; **e**, 12.5 μM; **f**, 25 μM; **g**, 50 μM; **h**, 100 μM) for various reaction times. Time points are 0, 0.5 min, 1.0 min, 2.0 min, 3.0 min, 4.5 min, 6 min, 8 min, 10 min, 13 min, 16 min, 20 min, 25 min and 30 min (left to right). The oligonucleotides (primer1, primer1+1, primer1+2 and primer1+3) were used as size markers in various gels. **i** and **j**. Kinetic curves of 8-oxo-dGTP incorporation opposite A.

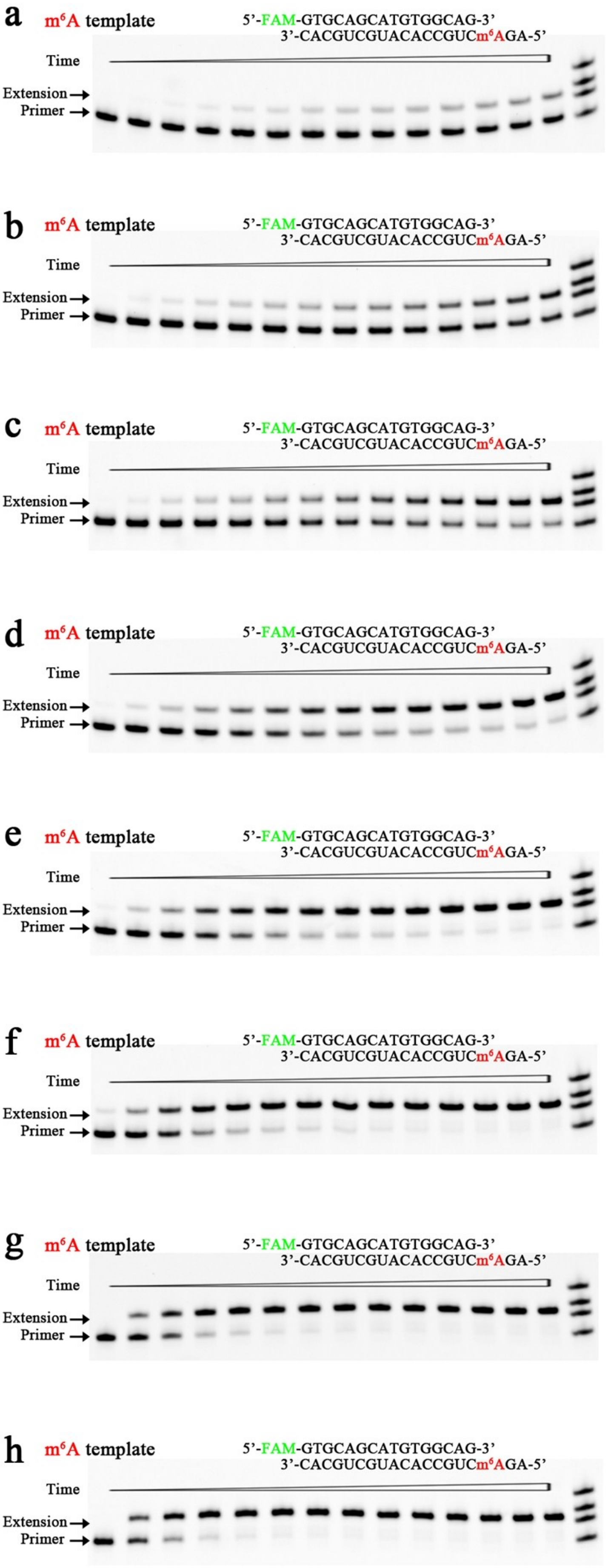
a
m6A template
5'-FAM-GTGCAGCATGTGGCAG-3'
3'-CACGUCGUACACCGUCm6AGA-5'
Time
Extension
Primer
b
m6A template
5'-FAM-GTGCAGCATGTGGCAG-3'
3'-CACGUCGUACACCGUCm6AGA-5'
Time
Extension
Primer
c
m6A template
5'-FAM-GTGCAGCATGTGGCAG-3'
3'-CACGUCGUACACCGUCm6AGA-5'
Time
Extension
Primer
d
m6A template
5'-FAM-GTGCAGCATGTGGCAG-3'
3'-CACGUCGUACACCGUCm6AGA-5'
Time
Extension
Primer
e
m6A template
5'-FAM-GTGCAGCATGTGGCAG-3'
3'-CACGUCGUACACCGUCm6AGA-5'
Time
Extension
Primer
f
m6A template
5'-FAM-GTGCAGCATGTGGCAG-3'
3'-CACGUCGUACACCGUCm6AGA-5'
Time
Extension
Primer
g
m6A template
5'-FAM-GTGCAGCATGTGGCAG-3'
3'-CACGUCGUACACCGUCm6AGA-5'
Time
Extension
Primer
h
m6A template
5'-FAM-GTGCAGCATGTGGCAG-3'
3'-CACGUCGUACACCGUCm6AGA-5'
Time
Extension
Primer

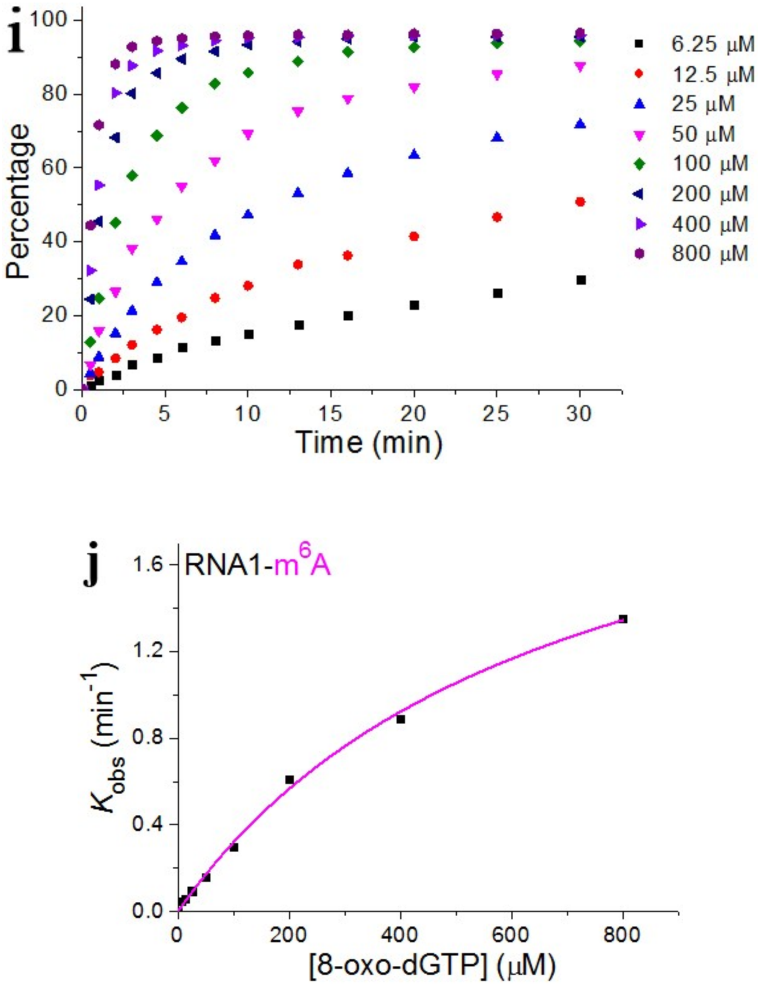

Figure S7. Pre-steady-state kinetics of 8-oxo-dGTP incorporation opposite RNA m6A by M-MuLV RT. Image of representative gels is shown here. Reactions were carried out as described in 'Materials and Methods' section. **a**-**h**, Preincubated M-MuLV RT and 50 nM primer/template duplex were mixed with various concentrations of 8-oxo-dGTP (**a**, 6.25 μM; **b**, 12.5 μM; **c**, 25 μM;□**d**, 50 μM; **e**, 100 μM; **f**, 200 μM; **g**, 400 μM; **h**, 800 μM) for various reaction times. Time points are 0, 0.5 min, 1.0 min, 2.0 min, 3.0 min, 4.5 min, 6 min, 8 min, 10 min, 13 min, 16 min, 20 min, 25 min and 30 min (left to right). The oligonucleotides (primer1, primer1+1, primer1+2 and primer1+3) were used as size markers in various gels. **i** and **j**. Kinetic curves of 8-oxo-dGTP incorporation opposite m6A.

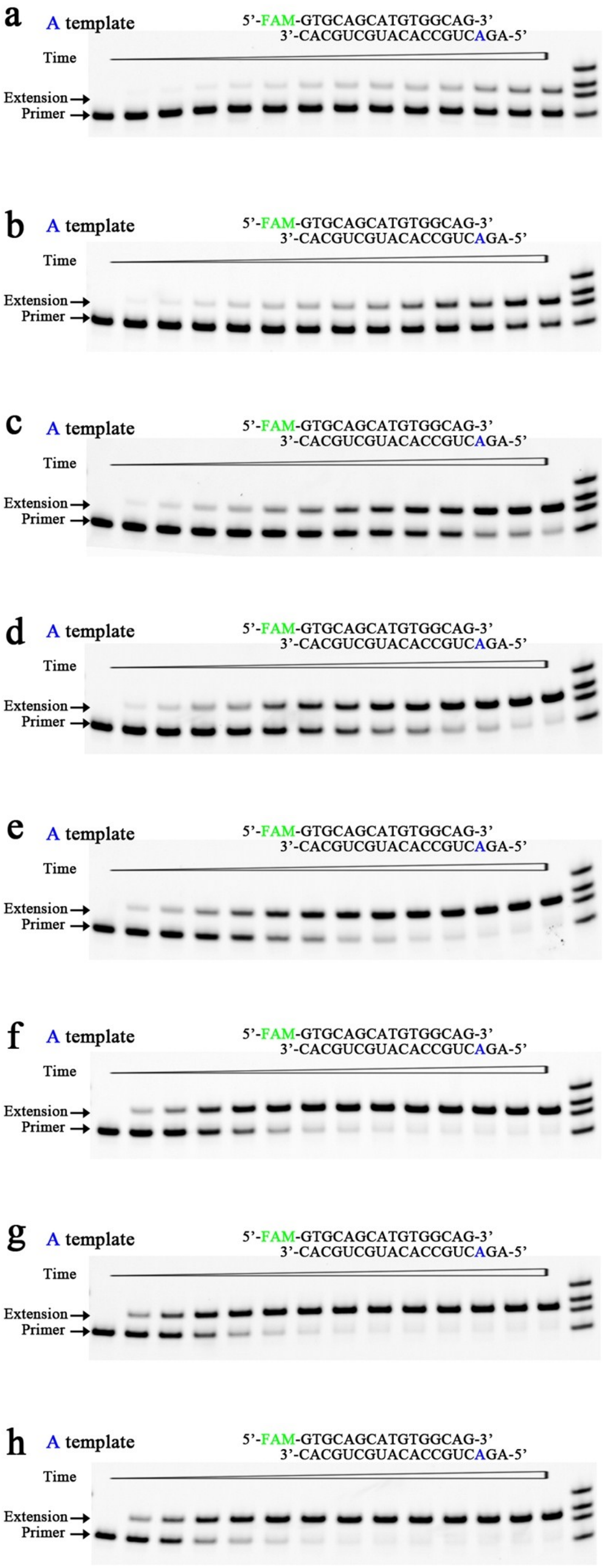
a
A template
5'-FAM-GTGCAGCATGTGGCAG-3'
3'-CACGUCGUACACCGUCAGA-5'
Time
Extension
Primer
b
A template
5'-FAM-GTGCAGCATGTGGCAG-3'
3'-CACGUCGUACACCGUCAGA-5'
Time
Extension
Primer
c
A template
5'-FAM-GTGCAGCATGTGGCAG-3'
3'-CACGUCGUACACCGUCAGA-5'
Time
Extension
Primer
d
A template
5'-FAM-GTGCAGCATGTGGCAG-3'
3'-CACGUCGUACACCGUCAGA-5'
Time
Extension
Primer
e
A template
5'-FAM-GTGCAGCATGTGGCAG-3'
3'-CACGUCGUACACCGUCAGA-5'
Time
Extension
Primer
f
A template
5'-FAM-GTGCAGCATGTGGCAG-3'
3'-CACGUCGUACACCGUCAGA-5'
Time
Extension
Primer
g
A template
5'-FAM-GTGCAGCATGTGGCAG-3'
3'-CACGUCGUACACCGUCAGA-5'
Time
Extension
Primer
h
A template
5'-FAM-GTGCAGCATGTGGCAG-3'
3'-CACGUCGUACACCGUCAGA-5'
Time
Extension
Primer

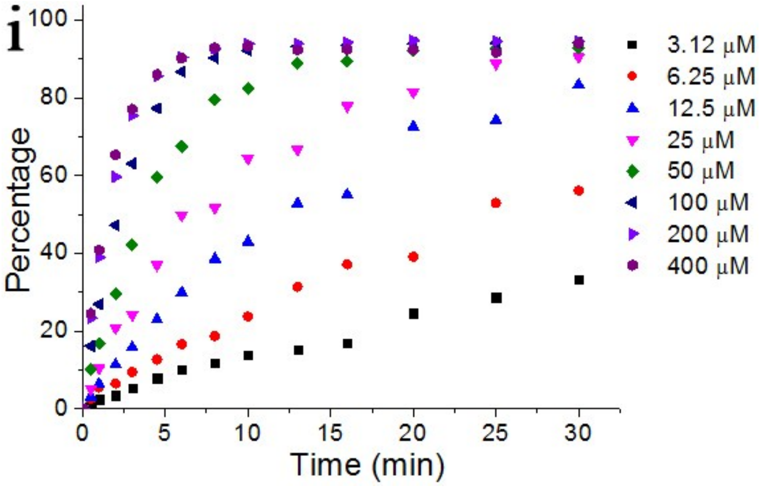

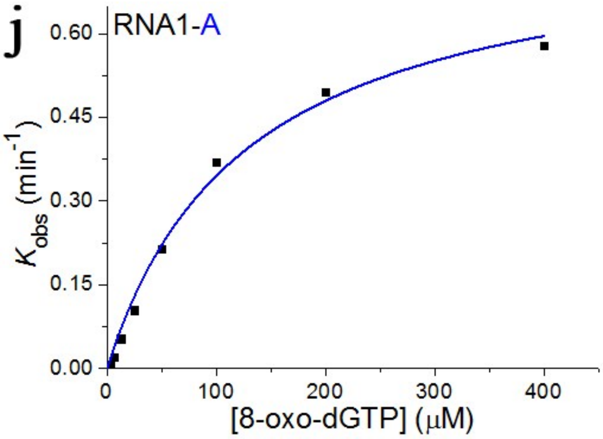

Figure S8. Pre-steady-state kinetics of 8-oxo-dGTP incorporation opposite RNA A by PS RT. Image of representative gels is shown here. Reactions were carried out as described in 'Materials and Methods' section. **a**-**h**, Preincubated PS RT and 50 nM primer/template duplex were mixed with various concentrations of 8-oxo-dGTP (**a**, 3.12 μM; **b**, 6.25 μM; **c**, 12.5 μM; **d**, 25 μM; **e**, 50 μM; **f**, 100 μM; **g**, 200 μM; **h**, 400 μM) for various reaction times. Time points are 0, 0.5 min, 1.0 min, 2.0 min, 3.0 min, 4.5 min, 6 min, 8 min, 10 min, 13 min, 16 min, 20 min, 25 min and 30 min (left to right). The oligonucleotides (primer1, primer1+1, primer1+2 and primer1+3) were used as size markers in various gels. **i** and **j**. Kinetic curves of 8-oxo-dGTP incorporation opposite A.

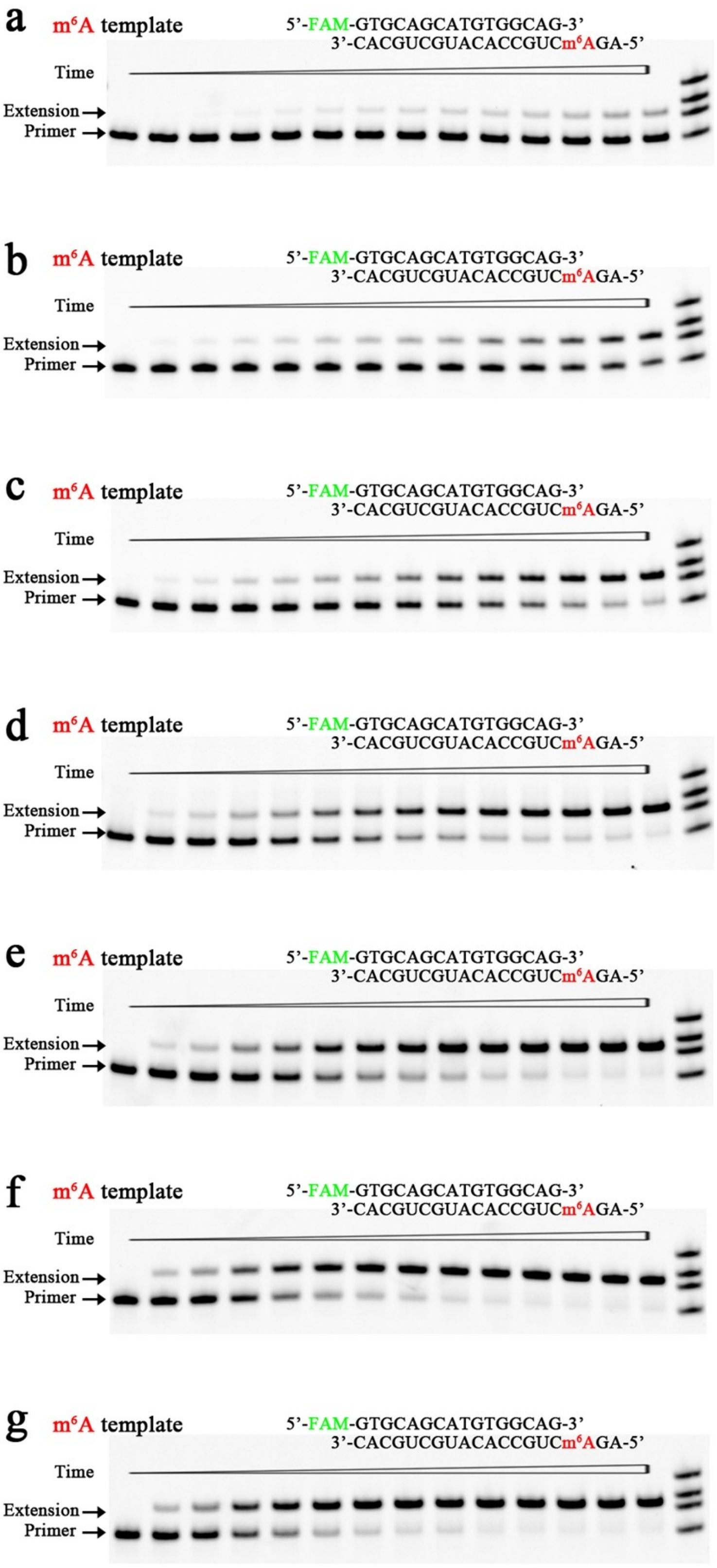
a
m6A template
5'-FAM-GTGCAGCATGTGGCAG-3'
3'-CACGUCGUACACCGUCm6AGA-5'
Time
Extension
Primer
b
m6A template
5'-FAM-GTGCAGCATGTGGCAG-3'
3'-CACGUCGUACACCGUCm6AGA-5'
Time
Extension
Primer
c
m6A template
5'-FAM-GTGCAGCATGTGGCAG-3'
3'-CACGUCGUACACCGUCm6AGA-5'
Time
Extension
Primer
d
m6A template
5'-FAM-GTGCAGCATGTGGCAG-3'
3'-CACGUCGUACACCGUCm6AGA-5'
Time
Extension
Primer
e
m6A template
5'-FAM-GTGCAGCATGTGGCAG-3'
3'-CACGUCGUACACCGUCm6AGA-5'
Time
Extension
Primer
f
m6A template
5'-FAM-GTGCAGCATGTGGCAG-3'
3'-CACGUCGUACACCGUCm6AGA-5'
Time
Extension
Primer
g
m6A template
5'-FAM-GTGCAGCATGTGGCAG-3'
3'-CACGUCGUACACCGUCm6AGA-5'
Time
Extension
Primer

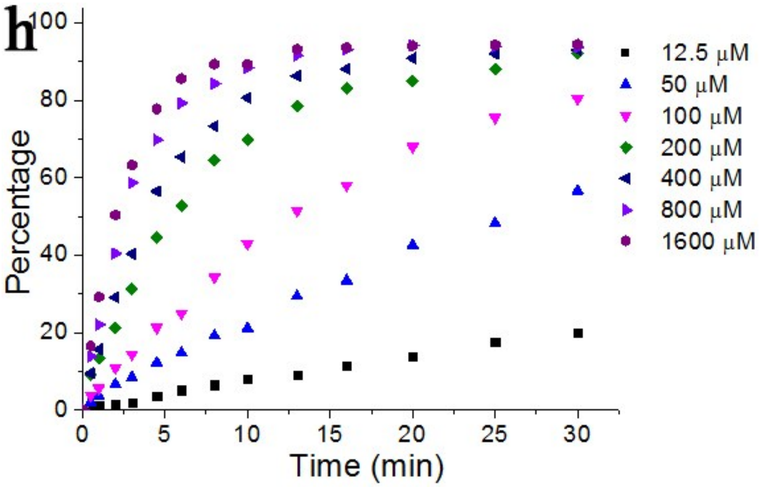

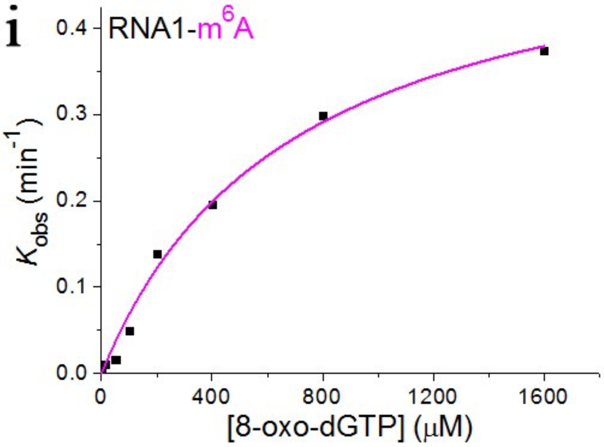

Figure S9. Pre-steady-state kinetics of 8-oxo-dGTP incorporation opposite RNA m^{6}A by PS RT. Image of representative gels is shown here. Reactions were carried out as described in 'Materials and Methods' section. **a**-**g**, Preincubated PS RT and 50 nM primer/template duplex were mixed with various concentrations of 8-oxo-dGTP (**a**, 12.5 μM; **b**, 50 μM; **c**, 100 μM; **d**, 200 μM; **e**, 400 μM; **f**, 800 μM; **g**, 1600 μM) for various reaction times. Time points are 0, 0.5 min, 1.0 min, 2.0 min, 3.0 min, 4.5 min, 6 min, 8 min, 10 min, 13 min, 16 min, 20 min, 25 min and 30 min (left to right). The oligonucleotides (primer1, primer1+1, primer1+2 and primer1+3) were used as size markers in various gels. **h** and **i**. Kinetic curves of 8-oxo-dGTP incorporation opposite m^{6}A.

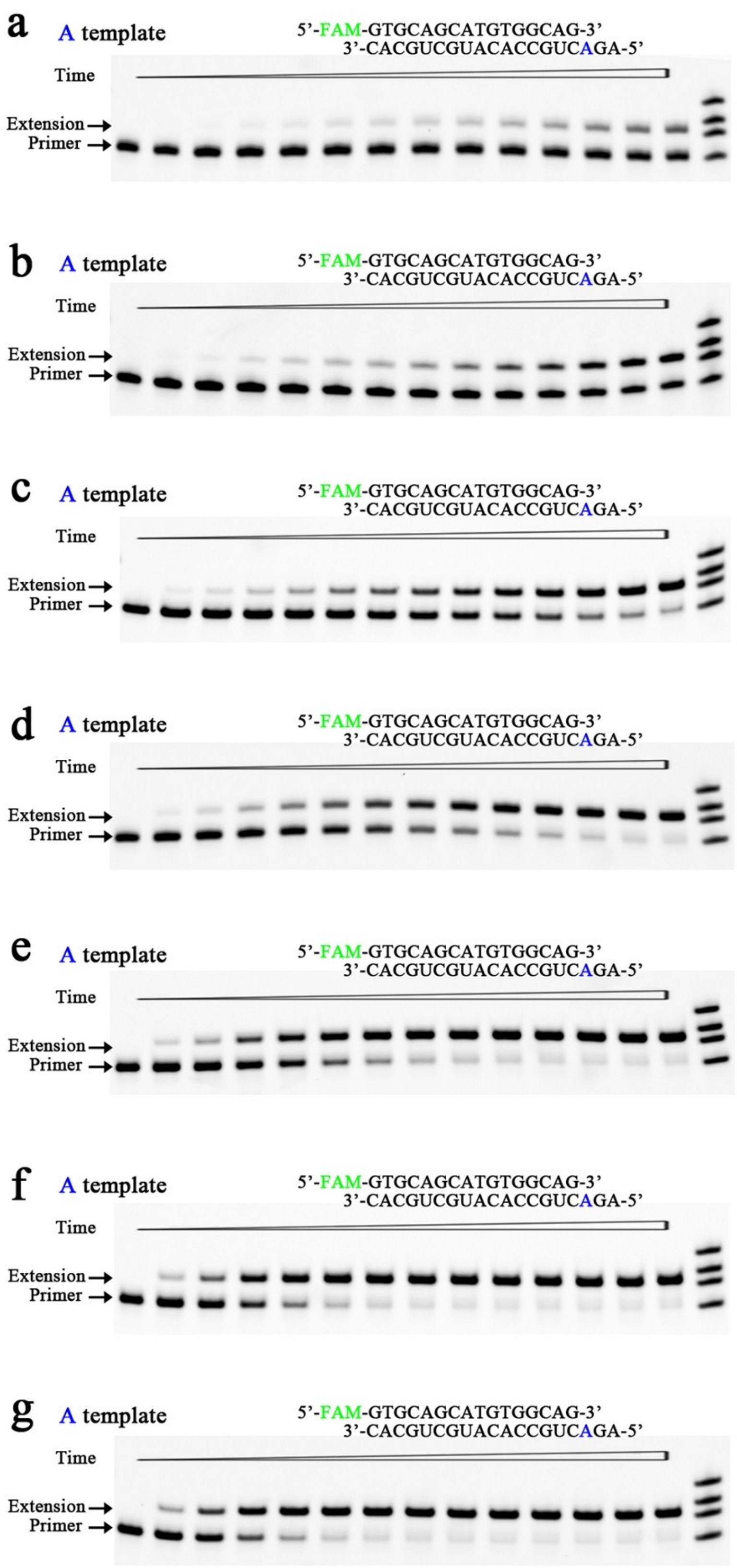
a
A template
5'-FAM-GTGCAGCATGTGGCAG-3'
3'-CACGUCGUACACCGUCAGA-5'
Time
Extension
Primer
b
A template
5'-FAM-GTGCAGCATGTGGCAG-3'
3'-CACGUCGUACACCGUCAGA-5'
Time
Extension
Primer
c
A template
5'-FAM-GTGCAGCATGTGGCAG-3'
3'-CACGUCGUACACCGUCAGA-5'
Time
Extension
Primer
d
A template
5'-FAM-GTGCAGCATGTGGCAG-3'
3'-CACGUCGUACACCGUCAGA-5'
Time
Extension
Primer
e
A template
5'-FAM-GTGCAGCATGTGGCAG-3'
3'-CACGUCGUACACCGUCAGA-5'
Time
Extension
Primer
f
A template
5'-FAM-GTGCAGCATGTGGCAG-3'
3'-CACGUCGUACACCGUCAGA-5'
Time
Extension
Primer
g
A template
5'-FAM-GTGCAGCATGTGGCAG-3'
3'-CACGUCGUACACCGUCAGA-5'
Time
Extension
Primer

h

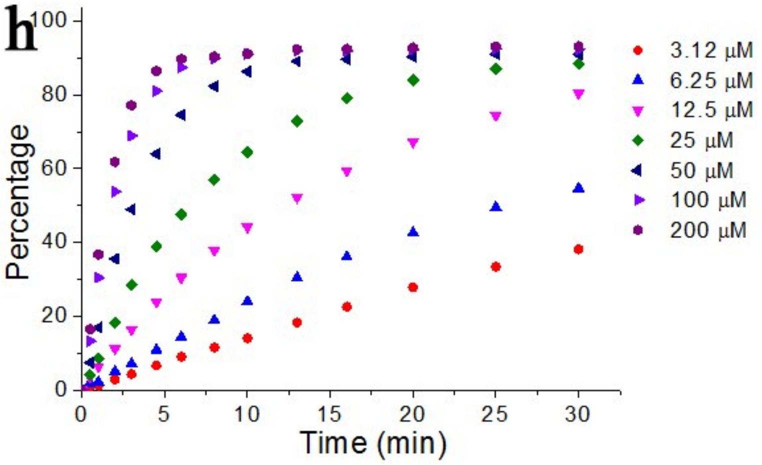

i

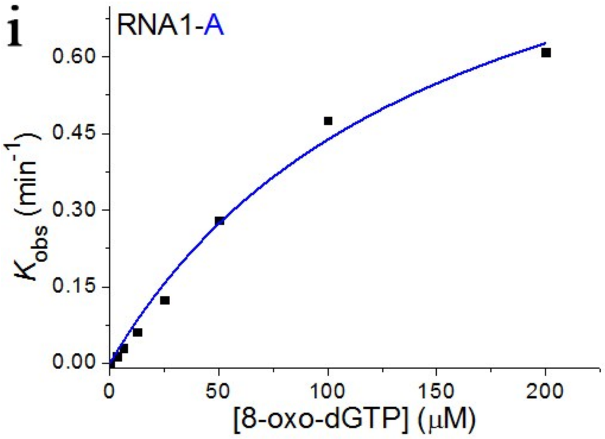

Figure S10. Pre-steady-state kinetics of 8-oxo-dGTP incorporation opposite RNA A by *Bst* DNA pol. Image of representative gels is shown here. Reactions were carried out as described in 'Materials and Methods' section. **a-g**, Preincubated *Bst* DNA pol and 50 nM primer/template duplex (DNA/RNA) were mixed with various concentrations of 8-oxo-dGTP (**a**, 3.12 μM; **b**, 6.25 μM; **c**, 12.5 μM; **d**, 25 μM; **e**, 50 μM; **f**, 100 μM; **g**, 200 μM) for various reaction times. Time points are 0, 0.5 min, 1.0 min, 2.0 min, 3.0 min, 4.5 min, 6 min, 8 min, 10 min, 13 min, 16 min, 20 min, 25 min and 30 min (left to right). The oligonucleotides (primer1, primer1+1, primer1+2 and primer1+3) were used as size markers in various gels. **h** and **i**. Kinetic curves of 8-oxo-dGTP incorporation opposite A.

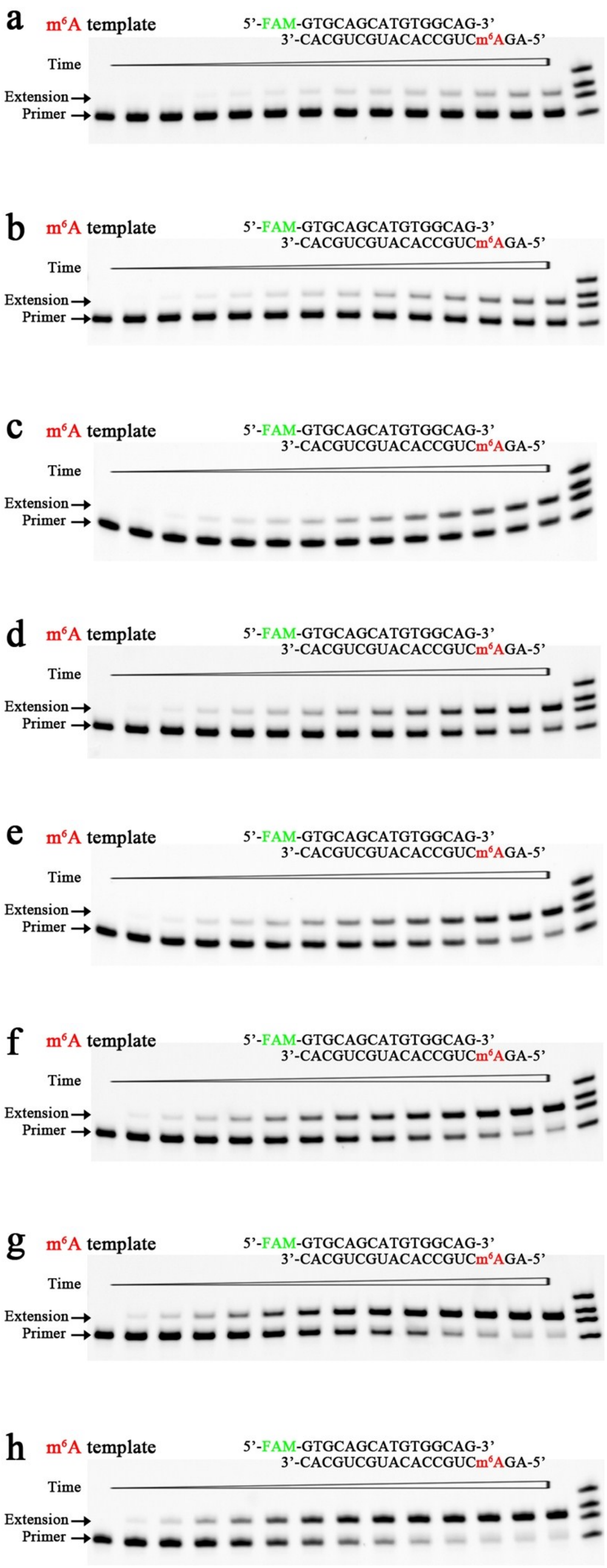
a
m6A template
5'-FAM-GTGCAGCATGTGGCAG-3'
3'-CACGUCGUACACCGUCm6AGA-5'
Time
Extension
Primer
b
m6A template
5'-FAM-GTGCAGCATGTGGCAG-3'
3'-CACGUCGUACACCGUCm6AGA-5'
Time
Extension
Primer
c
m6A template
5'-FAM-GTGCAGCATGTGGCAG-3'
3'-CACGUCGUACACCGUCm6AGA-5'
Time
Extension
Primer
d
m6A template
5'-FAM-GTGCAGCATGTGGCAG-3'
3'-CACGUCGUACACCGUCm6AGA-5'
Time
Extension
Primer
e
m6A template
5'-FAM-GTGCAGCATGTGGCAG-3'
3'-CACGUCGUACACCGUCm6AGA-5'
Time
Extension
Primer
f
m6A template
5'-FAM-GTGCAGCATGTGGCAG-3'
3'-CACGUCGUACACCGUCm6AGA-5'
Time
Extension
Primer
g
m6A template
5'-FAM-GTGCAGCATGTGGCAG-3'
3'-CACGUCGUACACCGUCm6AGA-5'
Time
Extension
Primer
h
m6A template
5'-FAM-GTGCAGCATGTGGCAG-3'
3'-CACGUCGUACACCGUCm6AGA-5'
Time
Extension
Primer

i

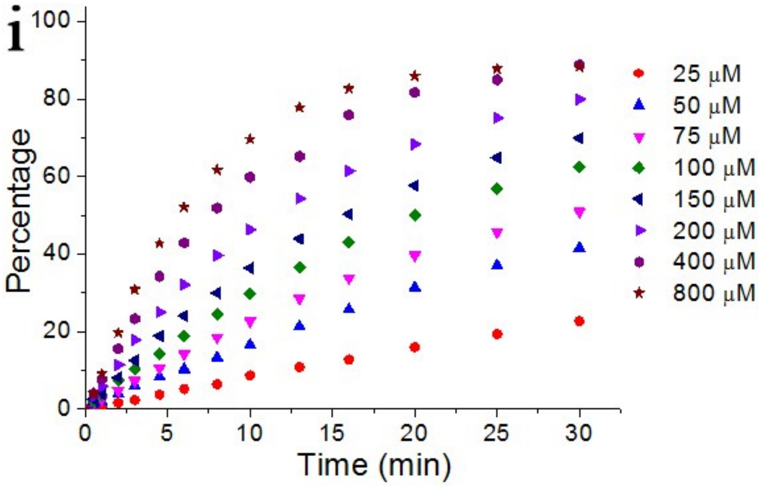

j

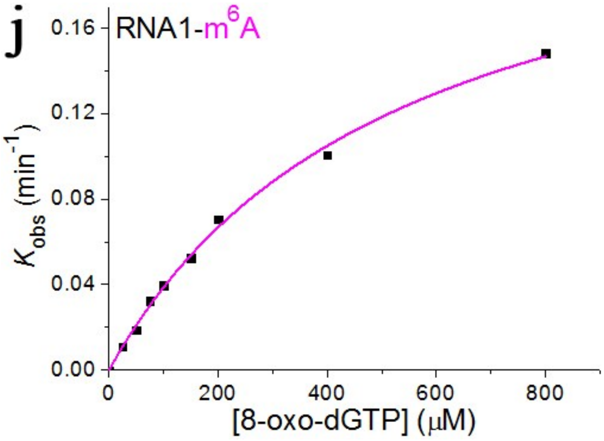

Figure S11. Pre-steady-state kinetics of 8-oxo-dGTP incorporation opposite RNA m^{6}A by *Bst* DNA pol. Image of representative gels is shown here. Reactions were carried out as described in 'Materials and Methods' section. **a**-**h**, Preincubated *Bst* DNA pol and 50 nM primer/template duplex (DNA/RNA) were mixed with various concentrations of 8-oxo-dGTP (**a**, 25 μM; **b**, 50 μM; **c**, 75 μM; **d**, 100 μM;□**e**, 150 μM; **f**, 200 μM; **g**, 400 μM; **h**, 800 μM) for various reaction times. Time points are 0, 0.5 min, 1.0 min, 2.0 min, 3.0 min, 4.5 min, 6 min, 8 min, 10 min, 13 min, 16 min, 20 min, 25 min and 30 min (left to right). The oligonucleotides (primer1, primer1+1, primer1+2 and primer1+3) were used as size markers in various gels. **i** and **j**. Kinetic curves of 8-oxo-dGTP incorporation opposite m^{6}A.

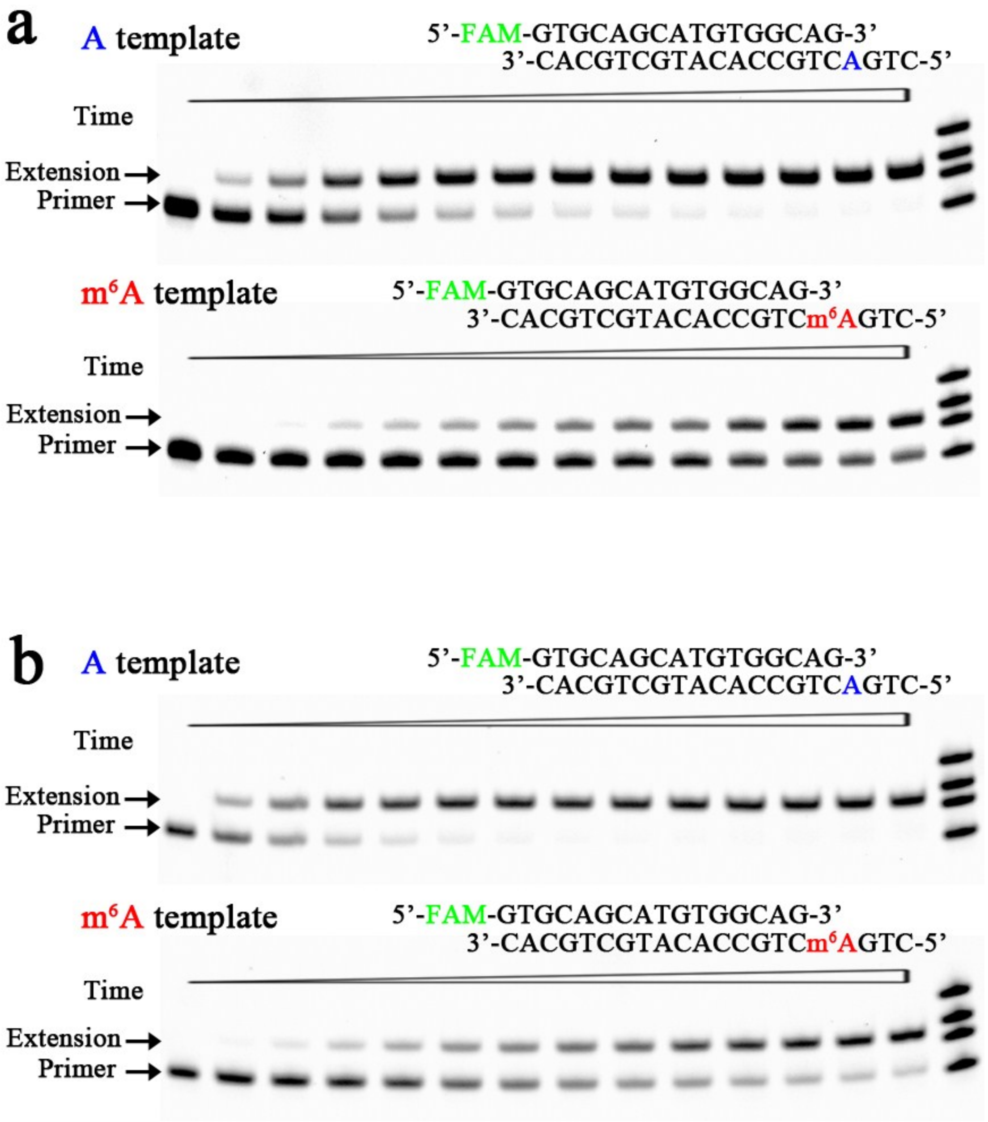

Figure S12. Impeded 8-oxo-dGTP incorporation by human DNA pol β opposite m^{6}A relative to A. Image of representative gels is shown here. Preincubated human DNA pol β and 50 nM primer/template duplex were mixed with various concentrations of 8-oxo-dGTP (**a**, 50 μM; **b**, 100 μM) for various reaction times. Time points are 0, 0.5 min, 1.0 min, 2.0 min, 3.0 min, 4.5 min, 6 min, 8 min, 10 min, 13 min, 16 min, 20 min, 25 min and 30 min (left to right). Reactions were carried out as described in 'Materials and Methods' section. The oligonucleotides (primer1, primer1+1, primer1+2 and primer1+3) were used as size markers in various gels.

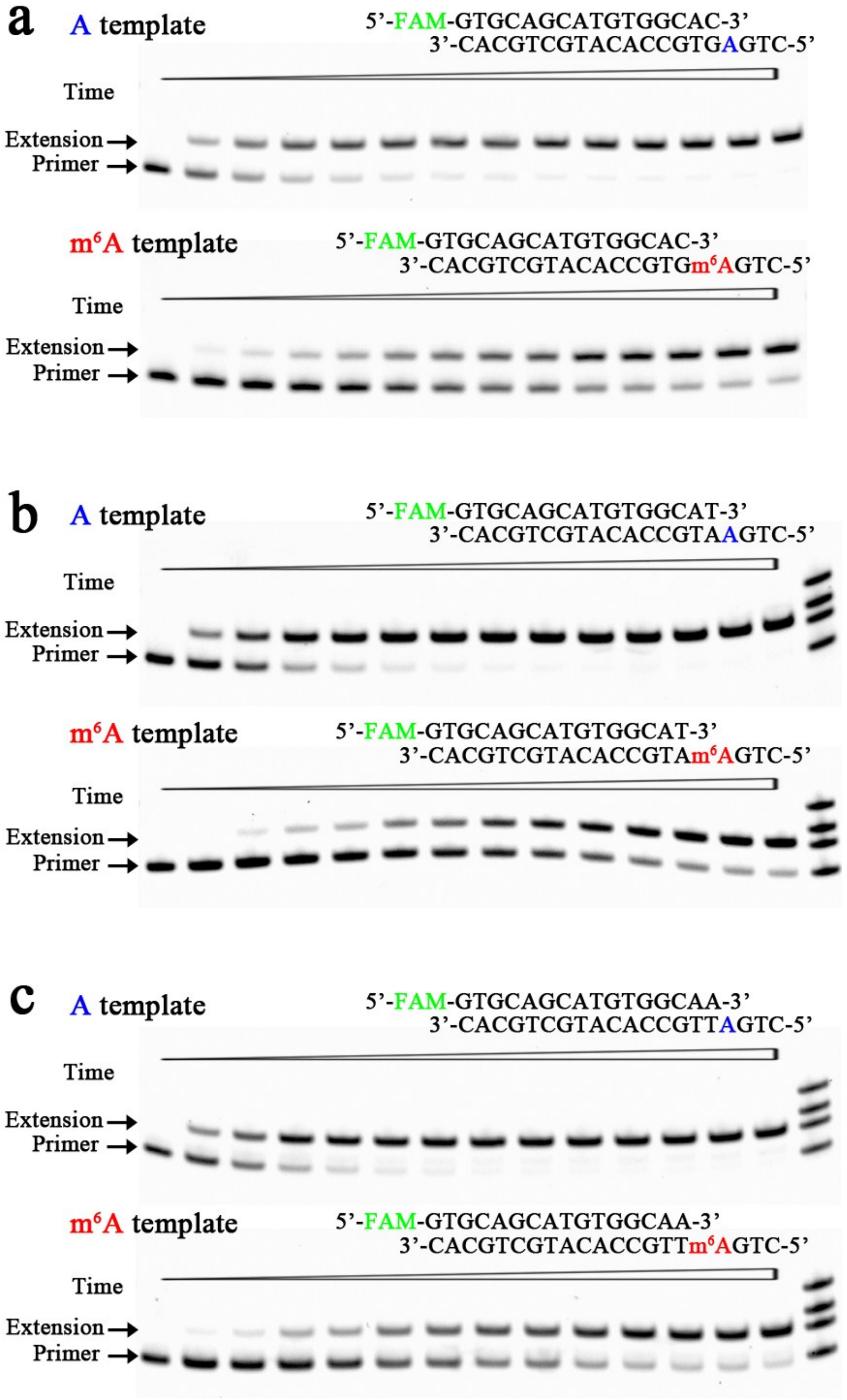

Figure S13. Impeded 8-oxo-dGTP incorporation by human DNA pol β opposite m^6A relative to A. Image of representative gels is shown here. Reactions were carried out as described in 'Materials and Methods' section using 50 nM primer/template duplex and 100 μM 8-oxo-dGTP (**a**, scaffold 2; **b**, scaffold 3; **c**, scaffold 4) for various reaction times. Time points are 0, 0.5 min, 1.0 min, 2.0 min, 3.0 min, 4.5 min, 6 min, 8 min, 10 min, 13 min, 16 min, 20 min, 25 min and 30 min (left to right). The oligonucleotides (primer1, primer1+1, primer1+2 and primer1+3) were used as size markers in various gels.

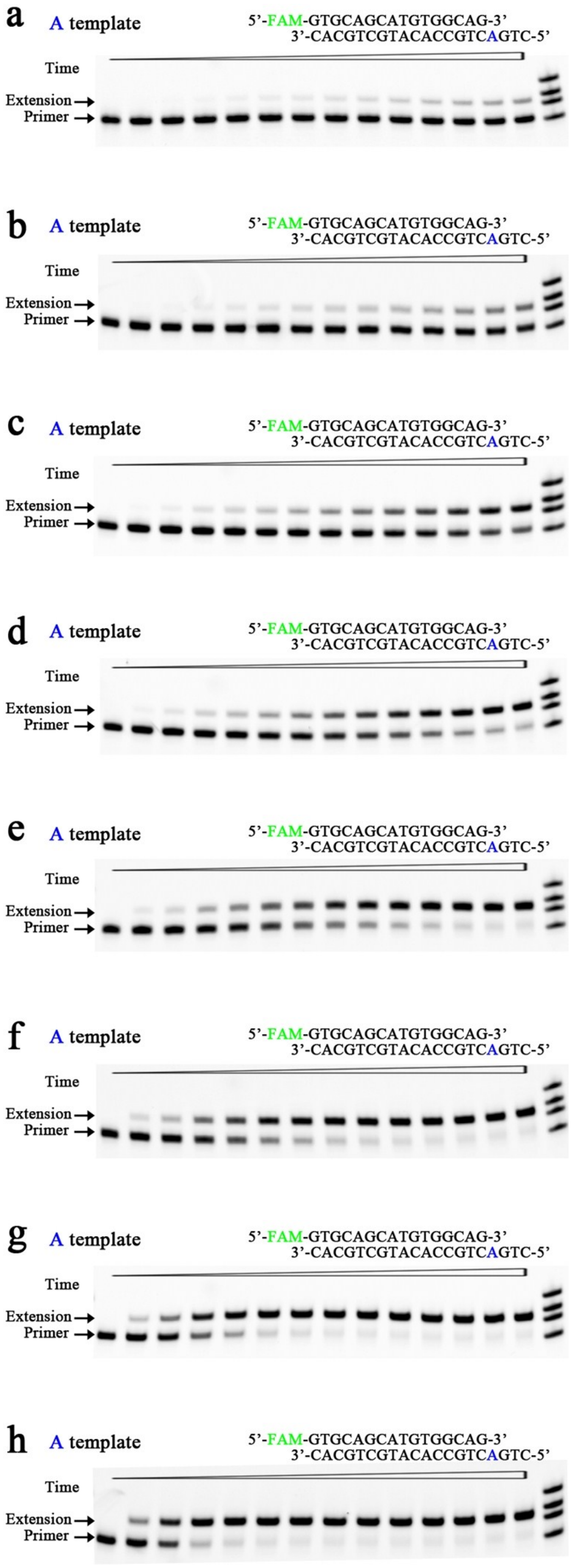
a
A template
5'-FAM-GTGCAGCATGTGGCAG-3'
3'-CACGTCGTACACCGTCAGTC-5'
Time
Extension
Primer
b
A template
5'-FAM-GTGCAGCATGTGGCAG-3'
3'-CACGTCGTACACCGTCAGTC-5'
Time
Extension
Primer
c
A template
5'-FAM-GTGCAGCATGTGGCAG-3'
3'-CACGTCGTACACCGTCAGTC-5'
Time
Extension
Primer
d
A template
5'-FAM-GTGCAGCATGTGGCAG-3'
3'-CACGTCGTACACCGTCAGTC-5'
Time
Extension
Primer
e
A template
5'-FAM-GTGCAGCATGTGGCAG-3'
3'-CACGTCGTACACCGTCAGTC-5'
Time
Extension
Primer
f
A template
5'-FAM-GTGCAGCATGTGGCAG-3'
3'-CACGTCGTACACCGTCAGTC-5'
Time
Extension
Primer
g
A template
5'-FAM-GTGCAGCATGTGGCAG-3'
3'-CACGTCGTACACCGTCAGTC-5'
Time
Extension
Primer
h
A template
5'-FAM-GTGCAGCATGTGGCAG-3'
3'-CACGTCGTACACCGTCAGTC-5'
Time
Extension
Primer

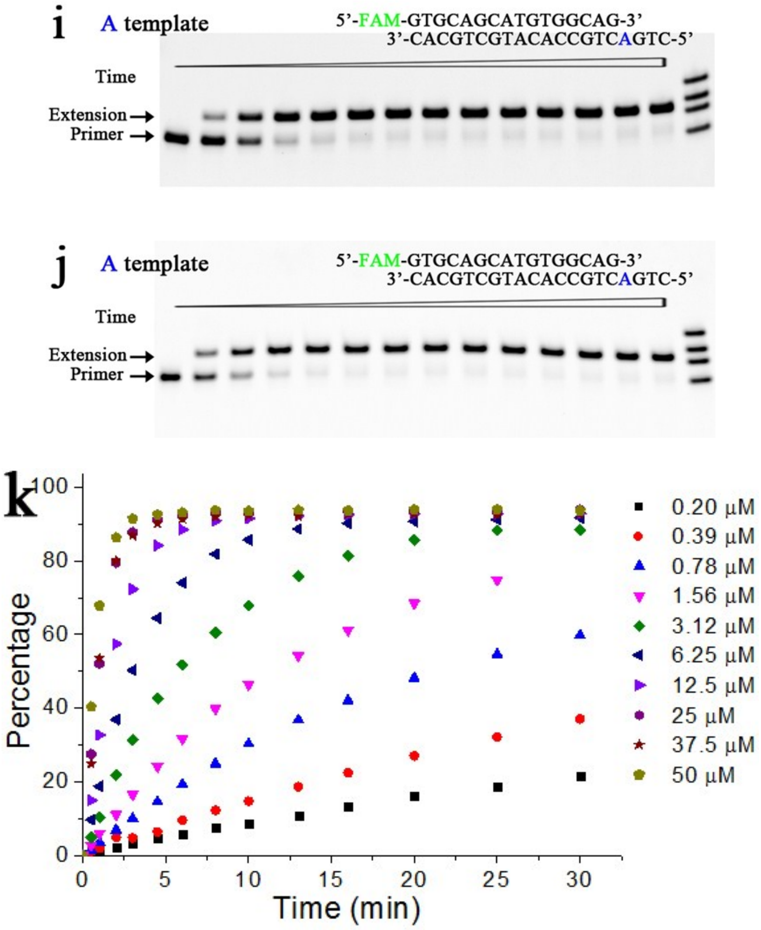

Figure S14. Pre-steady-state kinetics of 8-oxo-dGTP incorporation opposite DNA A by *Bst* DNA pol. Image of representative gels is shown here. Reactions were carried out as described in ‘Materials and Methods’ section. **a**-**i**, Preincubated *Bst* DNA pol and 50 nM primer/template duplex were mixed with various concentrations of 8-oxo-dGTP (**a**, 0.20 μM; **b**, 0.39 μM; **c**, 0.78 μM; **d**, 1.56 μM; **e**, 3.12 μM; **f**, 6.25 μM; **g**, 12.5 μM; **h**, 25 μM; **i**, 37.5 μM; **j**, 50 μM) for various reaction times. Time points are 0, 0.5 min, 1.0 min, 2.0 min, 3.0 min, 4.5 min, 6 min, 8 min, 10 min, 13 min, 16 min, 20 min, 25 min and 30 min (left to right). The oligonucleotides (primer1, primer1+1, primer1+2 and primer1+3) were used as size markers in various gels. **k**. Kinetic curves of 8-oxo-dGTP incorporation opposite DNA A.

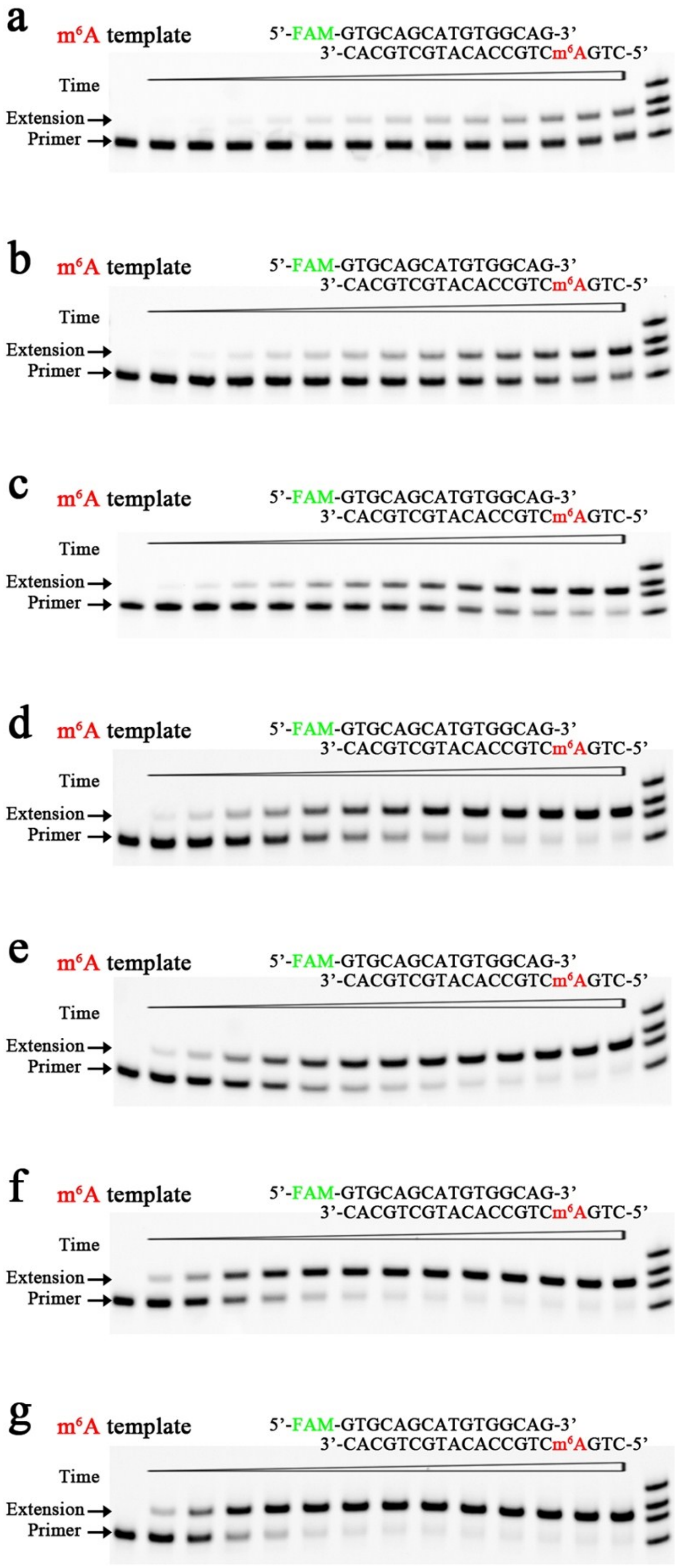
a
m6A template
5’-FAM-GTGCAGCATGTGGCAG-3’
3’-CACGTCGTACACCGTCm6AGTC-5’
Time
Extension
Primer
b
m6A template
5’-FAM-GTGCAGCATGTGGCAG-3’
3’-CACGTCGTACACCGTCm6AGTC-5’
Time
Extension
Primer
c
m6A template
5’-FAM-GTGCAGCATGTGGCAG-3’
3’-CACGTCGTACACCGTCm6AGTC-5’
Time
Extension
Primer
d
m6A template
5’-FAM-GTGCAGCATGTGGCAG-3’
3’-CACGTCGTACACCGTCm6AGTC-5’
Time
Extension
Primer
e
m6A template
5’-FAM-GTGCAGCATGTGGCAG-3’
3’-CACGTCGTACACCGTCm6AGTC-5’
Time
Extension
Primer
f
m6A template
5’-FAM-GTGCAGCATGTGGCAG-3’
3’-CACGTCGTACACCGTCm6AGTC-5’
Time
Extension
Primer
g
m6A template
5’-FAM-GTGCAGCATGTGGCAG-3’
3’-CACGTCGTACACCGTCm6AGTC-5’
Time
Extension
Primer

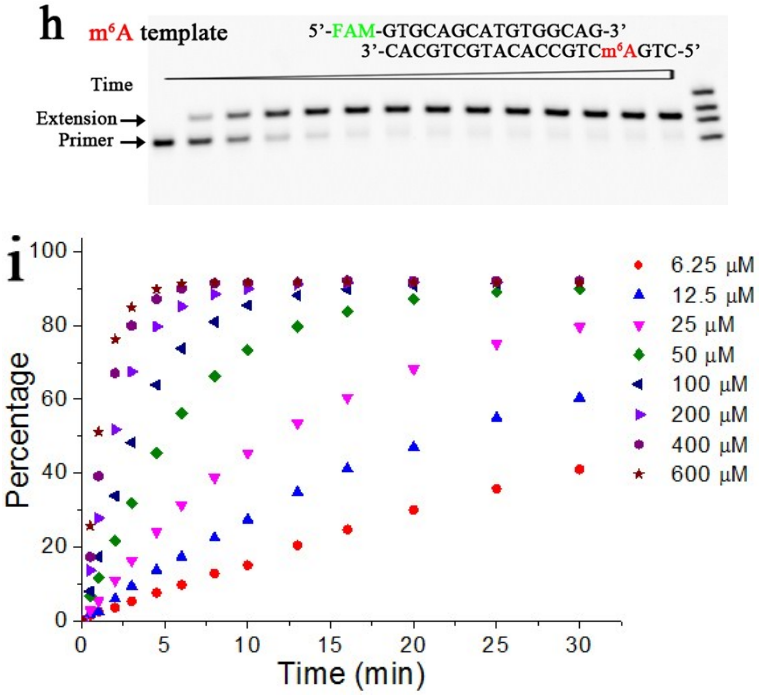

Figure S15. Pre-steady-state kinetics of 8-oxo-dGTP incorporation opposite DNA m^{6}A by *Bst* DNA pol. Image of representative gels is shown here. Reactions were carried out as described in 'Materials and Methods' section. **a**-**g**, Preincubated *Bst* DNA pol and 50 nM primer/template duplex were mixed with various concentrations of 8-oxo-dGTP (**a**, 6.25 μM; **b**, 12.5 μM; **c**, 25 μM; **d**, 50 μM; **e**, 100 μM; **f**, 200 μM; **g**, 400 μM; **h**, 600 μM) for various reaction times. Time points are 0, 0.5 min, 1.0 min, 2.0 min, 3.0 min, 4.5 min, 6 min, 8 min, 10 min, 13 min, 16 min, 20 min, 25 min and 30 min (left to right). The oligonucleotides (primer1, primer1+1, primer1+2 and primer1+3) were used as size markers in various gels. **i**. Kinetic curves of 8-oxo-dGTP incorporation opposite DNA m^{6}A.

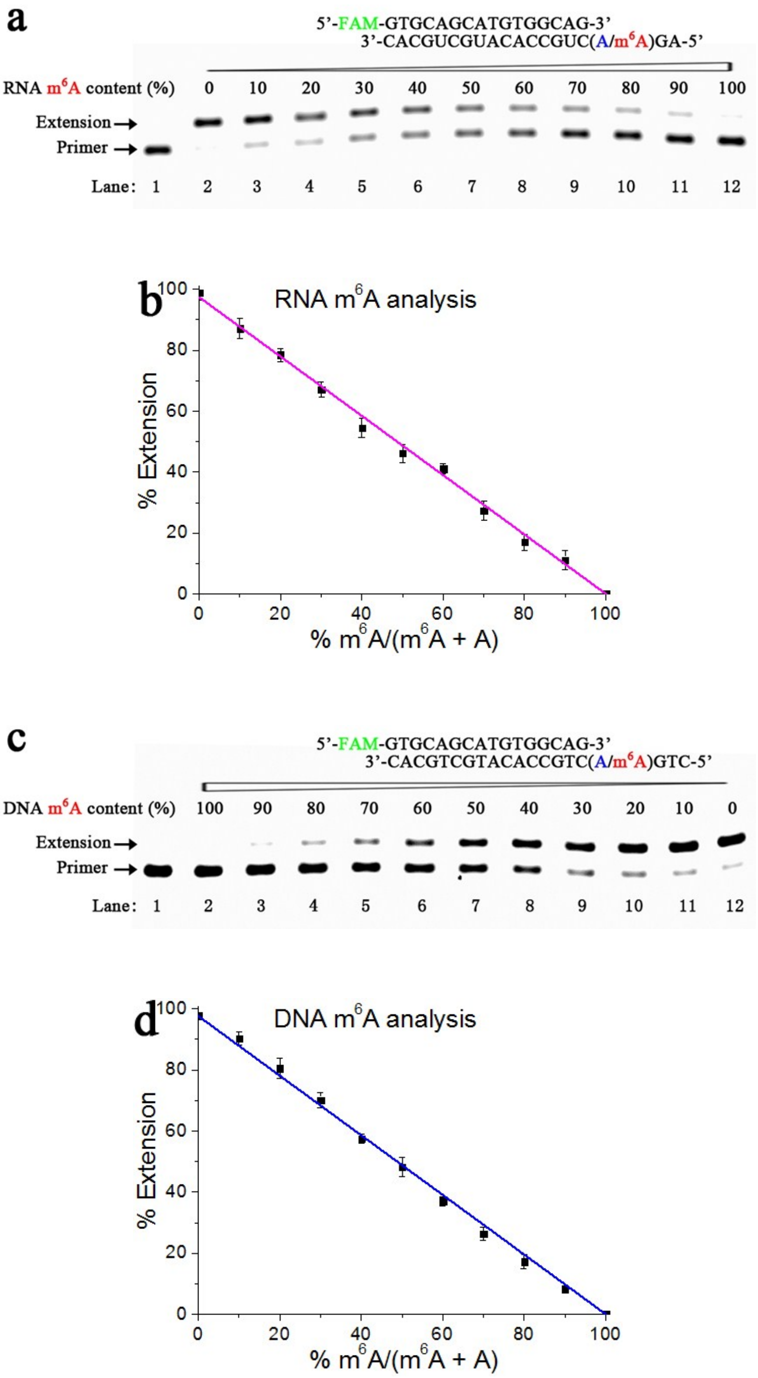

Figure S16. Quantitative analysis of the m^{6}A content of RNA or DNA samples. The extension condition is as following: 1.0 U *Bst* DNA polymerase and 10 μM 8-oxo-dGTP at 45 ^{o}C for 30 mins. **a**. lane 1, primer1 was used as a marker; lane 2 - lane 12, RNA templates with varied m^{6}A contents were analyzed. **b**. An inverse linear relationship between the fraction of extended primer and RNA m^{6}A content. **c**. lane 1, primer1 was used as a marker; lane 2 - lane 12, DNA templates with varied m^{6}A contents were analyzed. **d**. An inverse linear relationship between the fraction of extended primer and DNA m^{6}A content.

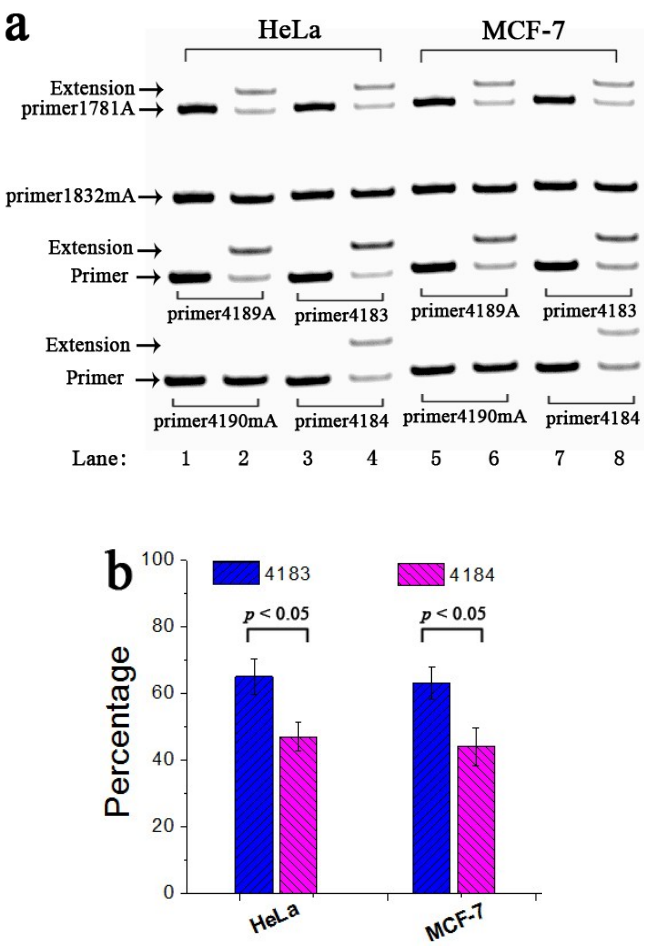

Figure S17. The m⁶A analysis of the 28S rRNA of cultured human cells. The extension condition is as following: 1.0 U *Bst* DNA polymerase and 10 μM 8-oxo-dGTP at 45 ^{o}C for 30 mins. **a**. lane 1, 3, 5 and 7, control samples without addition of 8-oxo-dGTP; lanes 2, 4, 6 and 8, analysis of the 28S rRNA of HeLa and MCF-7 cells. **b**. Percentage of 8-oxo-dGTP incorporation opposite position 4183 or 4184 of the 28S rRNA.
